# Affected energy metabolism under manganese stress governs cellular toxicity

**DOI:** 10.1038/s41598-017-12004-3

**Published:** 2017-09-19

**Authors:** Gursharan Kaur, Vineet Kumar, Amit Arora, Ajay Tomar, Ashish, Runa Sur, Dipak Dutta

**Affiliations:** 1 CSIR-Institute of Microbial Technology, Sector 39-A, Chandigarh, 160036 India; 2 Department of Biophysics, Molecular Biology & Bioinformatics, Calcutta University, Kolkata, India

## Abstract

Excessive manganese exposure is toxic, but a comprehensive biochemical picture of this assault is poorly understood. Whether oxidative stress or reduced energy metabolism under manganese exposure causes toxicity is still a debate. To address this, we chose Δ*mnt* *P* *Escherichia coli*, a highly manganese-sensitive strain, in this study. Combining microarray, proteomics, and biochemical analyses, we show that the chronic manganese exposure rewires diverse regulatory and metabolic pathways. Manganese stress affects protein and other macromolecular stability, and envelope biogenesis. Most importantly, manganese exposure disrupts both iron-sulfur cluster and heme-enzyme biogenesis by depleting cellular iron level. Therefore, the compromised function of the iron-dependent enzymes in the tricarboxylic acid cycle, and electron transport chain impede ATP synthesis, leading to severe energy deficiency. Manganese stress also evokes reactive oxygen species, inducing oxidative stress. However, suppressing oxidative stress does not improve oxidative phosphorylation and cell growth. On the contrary, iron supplementation resumed cell growth stimulating oxidative phosphorylation. Therefore, we hypothesize that affected energy metabolism is the primal cause of manganese toxicity.

## Introduction

Iron, the most abundant transition metal in biology, serves as a cationic cofactor or remains embedded in the iron-sulfur clusters (ISC) and heme groups of the reaction centers of proteins^1^. The major issue with the iron is that it leaches out from the protein complexes upon reacting with endogenous superoxide anions (O_2_^**−**^)^2^. Subsequent oxidation of this free metal by hydrogen peroxide (H_2_O_2_) generates highly toxic hydroxyl radical (∙OH) in the cell^2^. On the other hand, manganese relieves oxidative stress and iron starvation by activating manganese-superoxide dismutase (SodA) and preserving the function of iron-dependent enzymes and sustains DNA metabolism^3–6^. However, excess manganese is a potential hazard. Manganese arrests cell growth and division of *E. coli* ^7,8^. Manganese exposure reduces iron transport, and inhibits the final step of heme biogenesis in *E. coli* ^7–9^. Thus, supplementing iron, or deleting *fur*, a gene that encodes a repressor for iron import, rescues the manganese toxicity^7,8^, suggesting that manganese and iron homeostasis mechanisms are intricately intertwined.

MntR- and Fur-mediated transcription regulations in *E. coli* maintain manganese and iron homeostasis, respectively^1^. The crosstalk between the MntR and Fur regulons presumably balances the manganese and iron levels^7,8^. On the other hand, the mammalian cells transport manganese and iron by a common divalent metal ion transporter (DMT1)^10–12^. Competitive uptake of one metal ion by DMT1 transporter in mammalian cells apparently interfere with the uptake and function of the other^13^. Thus, excessive exposure of manganese can disrupt iron homeostasis, reducing iron metabolism. It is interesting that despite the huge gap in compositional and regulatory entities, evolution has conserved the above-mentioned relationship between manganese and iron levels in the cell. Iron depletion is associated with the increased manganese levels in the rat brain^12,14,15^. In humans, excess manganese affects the metabolic processes in iron-rich basal ganglia, resulting in neurodegenerative disorders similar to Parkinson’s disease (PD)^16,17^. Many reports point to oxidative stress evoked under manganese stress could lead to toxicity^18–21^. On the contrary, a number of other studies indicate that inactivation of mitochondrial energy metabolism *in vitro* and *in vivo*, could be the cause of manganese toxicity^20–34^. However, the precise mechanism underlying the effects of manganese on biochemical pathways remains ambiguous.

*E. coli* MntP is an inner-membrane-bound manganese efflux pump^7,8^. Δ*mnt* *P* strain exhibits profound growth retardation when exposed to manganese, presumably due to an accumulation of manganese in the cytoplasm^7,8^. We took advantage of this phenomenon to find the route of manganese toxicity. We unravel that the iron depletion under manganese stress not only affects heme-protein biogenesis, as observed earlier^9^, but also disrupts the biogenesis and the function of ISC proteins. These observations, and other biochemical assays promoted us to hypothesize that the loss of both heme and ISC protein functions dramatically affects energy metabolism, which govern manganese toxicity in Δ*mnt* *P* strain of *E. coli*.

## Results

### Gene expression profile under manganese stress

**Figure 1:**
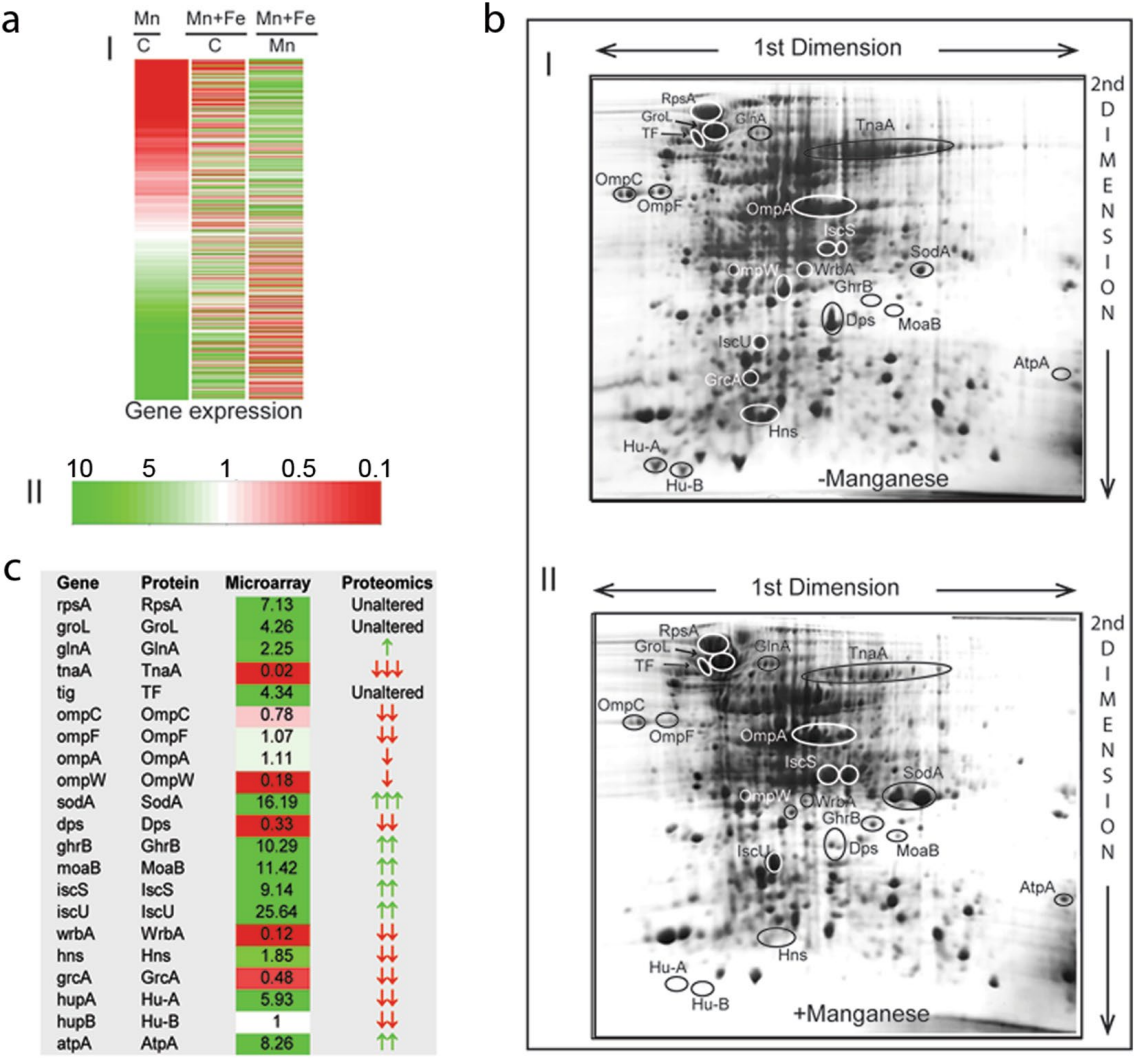
Correlation between microarray and proteomics data. **(a)** Heat-maps showing differential expressions for all transcribing genes (I) in the microarray. The color key (II) shows upregulation (green), downregulation (red) and indifferent expression (white) of the genes. The cluster Mn/C represents differential expressions in the manganese-fed cells versus control cells. Similarly, the cluster (Mn + Fe)/C represent differential expression of the genes in the presence of MnCl_2_ and FeCl_3_ versus control cells. The cluster (Mn + Fe)/Mn represent the degree of suppression of the manganese-induced differential expression by iron supplementation. (**b)** Proteins from the unstressed and manganese-stressed cells were separated in two 2D gels (panels I and II, respectively) to show the differential expressions. The labeled protein spots were identified by tandem MS analyses. (**c**) This panel summarizes the qualitative similarities and differences between mRNA and protein levels observed in the microarray and proteomics studies.

The microarray experiment was conducted using Agilent based single color platform (Fig. 1a). Considering a minimum of 3-fold differential expression, we identified 420 activated and 396 repressed genes. EcoCyc database and KEGG tools^1,35^ were used to group these genes under appropriate pathways (Table 1; Supplementary Tables S1–S8). Some other genes in these pathways, which show at least 2-fold differential expression, were also accounted in the pathway mapping (Supplementary Tables S1–S8). To validate the differential expressions, we performed quantitative PCR (q-PCR), targeting the mRNA levels of a few genes. The trends of gene expression revealed from q-PCR (Supplementary Fig. S1) matches with the microarray (Supplementary Tables S1–S8). Since iron supplementation suppresses manganese stress^7,8^, we also performed microarray in the presence of manganese and iron to identify the differential expressions of the genes (Fig. 1a).

**Table 1:** Summary of microarray expression profile.

Metabolisms	Genes and pathways
Protein metabolism, proteostasis and transport	∗Ribosome and its biogenesis
	∗rRNA and tRNA genes
	∗Chaperone, protease and protein translocation
DNA and RNA metabolism	∗Replication and cell division
	∗RNAP and associated genes
	#Two component system
Membrane biogenesis	∗Lipid and lipopolysaccharide
	∗Cell wall biogenesis
Energy Metabolism	∗Electron transport and ATP synthase genes
	∗Ubiquinone synthesis
	#Menaquinone synthesis
	#Anaerobic respiration and nitrogen metabolism
Carbon metabolism	∗Glycerol uptake and metabolism
	∗Pentose phosphate pathway
	∗Glycolysis
	#Sugar uptake genes
	#Dicarboxylate transport
Redox balance	∗Oxidative stress markers
	∗Redox cycle and cysteine metabolism
Iron metabolism	#Iron import and enterobactin synthesis
	∗ISC and heme biogenesis
Flagellar biogenesis and motility	#Flagellar structural genes
	#Flagellar assembly genes
	#Other motility related genes

The major genes and pathways, which were upregulated (∗) or downregulated (#) under manganese toxicity, are tabulated to show the affected metabolism.

Qualitatively, the two-dimensional (2D) proteomics revealed an overall correlation with the microarray trends, albeit with a few discrepancies (Fig. 1b and c). We observed that manganese stress activated chaperones and proteases genes (Supplementary Table S1). In corroboration, we show that manganese stress promoted protein misfolding and degradation (Supplementary Fig. S2). This phenomenon could be the reason for the discrepancies between microarray and proteomics expression profile.

### Manganese-induced iron depletion blocks ISC and heme protein biogenesis

**Figure 2:**
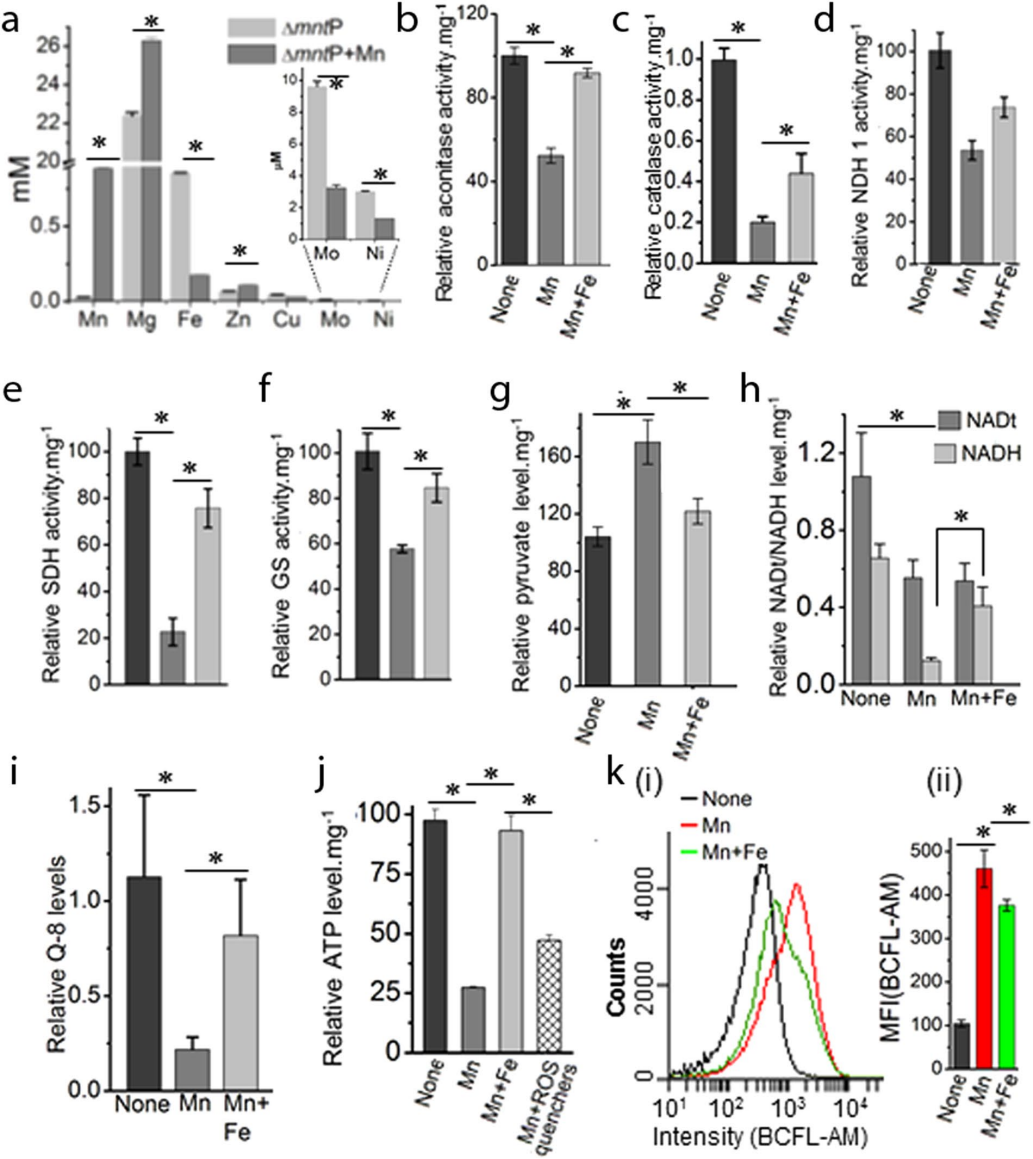
Manganese-induced iron deficiency blocks energy metabolism. **(a)** Manganese stress alters cellular levels of iron and many other metals. Extremely trace metals, Mo and Ni, are shown by zooming. (**b**–**f**) Manganese stress impairs aconitase, catalase, NDH-1, SDH and Glutamate synthase (GS) activities, while iron supplementation rescues enzyme the activities. (**g)** Pyruvate levels under manganese stress and iron supplementation were plotted as a function of normalized absorbance at 450 nm per milligram of cellular proteins. (**h)** Manganese stress reduces the cellular NAD and NADH levels. Interestingly, iron supplementation does not improve total NAD (NADt), but increases NADH levels. (**i**) The relative ubiquinone (Q-8) levels in the manganese fed cells and under iron supplementation were shown. (**j)**. The panel represents that manganese stress reduces cellular ATP level, while iron supplementation rescues it. (**k)**. (i) The FACS data represents the relative pH changes as a function of florescence intensity of BCFL-AM, a pH indicator dye. (ii) Normalized mean fluorescence intensities (MFI) from 3 different FACS are also plotted. The data in the above panels are mean ± SD (n = 3); *P < 0.001, paired T-test.

Manganese stress antagonizes iron metabolism, leading to iron depletion in *E. coli* ^7,9^ (Fig. 2a). The iron depletion appears to be the result of downregulation of the genes encoding iron import and enterobactin metabolizing proteins (Supplementary Table S2). Interestingly, iron storage protein (Dps) was also downregulated both transcriptionally and translationally under manganese stress (Fig. 1c). Consequently, activation of the genes (*isc*, *hsc*, *fdx* and *nfu*), which encode the enzymes required for the maturation of ISC-proteins, were observed (Supplementary Table S2). Particularly, *isc* operon (*isc*, *hsc*, *fdx* genes) and *nfu* are repressed by holo-IscR regulator^1^. Therefore, it appears that cellular iron scarcity produced ISC deficient apo-IscR that de-represses *isc* operon and *nfu* gene. Consistently, the proteomics data shows overexpression of IscS and IscU proteins under manganese stress (Fig. 1c). Apart from the ISC protein biogenesis, IscS function is involved in thiamine pyrophosphate (TPP), and molybdenum cofactor (Moco), and thiolated-tRNA synthesis^1^. Additionally, TPP, Moco and tRNA thiolation pathway genes (*thi*, *moa*, *mog*, *mnm*, *tus* and *dxs*) were also upregulated under manganese stress (Supplementary Table S2). Therefore, activation of these genes including *i* *scS* indicates that tRNA thiolation, and TPP and Moco syntheses would be affected under manganese stress. Particularly, molybdenum deficiency (Fig. 2a) and repression of molybdenum importer (*mod*) genes (Supplementary Table S2) directly indicated the declined Moco synthesis in the manganese-fed cells.

Manganese stress increases the level of protoporphyrin-IX reducing cellular heme levels^9^. The scarcity of Fe^2+^ under iron depletion (Fig. 2a) could hinder protoporphyrin-IX to ferroheme biogenesis by ferrochelatase enzyme (HemH), resulting in protoporphyrin-IX accumulation. Activation of some *hem* genes in the heme synthesis pathway were observed under Mn stress (Supplementary Table S2 and Supplementary Fig. S3).

### Mature ISC and heme protein deficiency affects energy metabolism

ISCs and heme are the integral parts of the electron transport chain (ETC) and tricarboxylic acid cycle (TCA) protein complexes^1^. For example, NADH dehydrogenase I (NDH-1), succinate dehydrogenase (SDH or SdhABCD), terminal oxidases, aconitases (AcnA and B) and fumarase (FumABC) enzymes extensively utilize these cofactors in the ETC and TCA cycle^1^. We demonstrate that manganese-fed cells exhibited the loss of aconitase activity (Fig. 2b). In addition, manganese stress reduced the activity of heme-containing catalase proteins (Fig. 2c). Since manganese stress did not alter the aconitase and catalase coding gene expressions (Supplementary Table S3), the loss of aconitase and catalase functions could be attributed to the disruption of ISC and heme biogenesis. Similarly, we have assessed the activities of NDH-1, SDH, and glutamate synthase (GS) that require ISC or Fe^2+^ for their function. All of these enzyme activities were declined significantly under manganese stress (Fig. 2d–f). Accordingly, exogenous iron supplementation rescued all of the above-mentioned enzyme activities (Fig. 2b–f).

Similarly, reduced ISC and heme protein biogenesis would also affect many other functions. Importantly, ISC-utilizing TCA cycle enzymes (AcnA, AcnB and SDH), which are important for the aerobic carbon flow and ATP synthesis, appeared to be blocked under manganese stress. This slow passage of carbon via TCA cycle increased relative pyruvate levels in the cells (Fig. 2g). As a result, manganese stress apparently modulated glycolytic carbon metabolism, repressing genes for energy rich hexose sugar uptake, and activating genes involved in the usage of energy poor glycerol and its derivatives (Supplementary Fig. S4 and Supplementary Table S4). Interestingly, previous studies suggest that manganese stress impairs oxidative hexose metabolism by inactivating glycolytic and TCA cycle enzymes in the astrocytes and other neuronal cells^30,31^. Overall, upregulation of the genes in the glycolysis and gluoconeogenesis, penose phosphate pathway (PPP) (*zwf*, *rpe*, *tal* *B*), pyridoxal phosphate (PLP) and ubiquinone synthesis pathways (*epd*, *pdx*, *dxs, ubi* and *isp* genes) could be the indicators of the affected energy metabolism under manganese stress (Supplementary Fig. S4 and Supplementary Table S4).

To show the oxygen consumption by the manganese-fed cells, we have used two indicator dyes. First, methylene blue sensor dye show that manganese stressed cells consume less dissolve oxygen than unstressed cells^36^ (Supplementary Fig. S5a). Second, Resazurin (7-Hydroxy-3*H*-phenoxazin-3-one 10-oxide) dye, which is purple or pink when cellular NADH/NADPH levels are low or high, respectively, indicated that aerobic respiration is dramatically decreased in the manganese-stressed cells^37^ (Supplementary Fig. S5b). Both of the dyes further indicated that iron supplementation rescues oxygen consumption to support aerobic respiration in the cells.

NDH-1 protein complexes utilize cellular NADH, which is generated by carbon metabolism, to initiate electron transport. The electrons are then shuttled between different ETC complexes and terminal oxidases via lipid soluble ubiquinone carriers. This process of electron transport simultaneously pumps out protons to generate a proton motive force (PMF) across the cell membrane for ATP synthesis^1^. We observed that manganese stress reduced total NAD (NADt) and NADH level in the cell (Fig. 2h). Besides, we show 5-fold reduction in total ubiquinone level in the manganese-fed cells (Fig. 2i). These observations indicate that ATP production under manganese stress could be affected. Therefore, using a luciferase-based assay, we demonstrated that manganese stress indeed reduced relative cellular ATP level (Fig. 2j). PMF also indirectly takes part in cellular pH homeostasis when extracytoplasmic protons enter via ATP synthase and flagellar motors^38^. Using cell penetrable pH sensor fluorescent dye, BCFL-AM, we found that manganese toxicity raised the relative cellular pH (Fig. 2k), indicating that weak PMF generated in the manganese-fed cells failed to maintain pH homeostasis. Conversely, supplementation of iron restores NADH and ubiquinone levels, thereby resumes ATP production and cellular pH (Fig. 2h–k). Interestingly, iron could not reverse the NADt level in the manganese-fed cells (Fig. 2h), indicating that iron only promoted NAD to NADH production by accelerating carbon metabolism.

### Reduced ETC function evokes ROS under manganese stress

**Figure 3:**
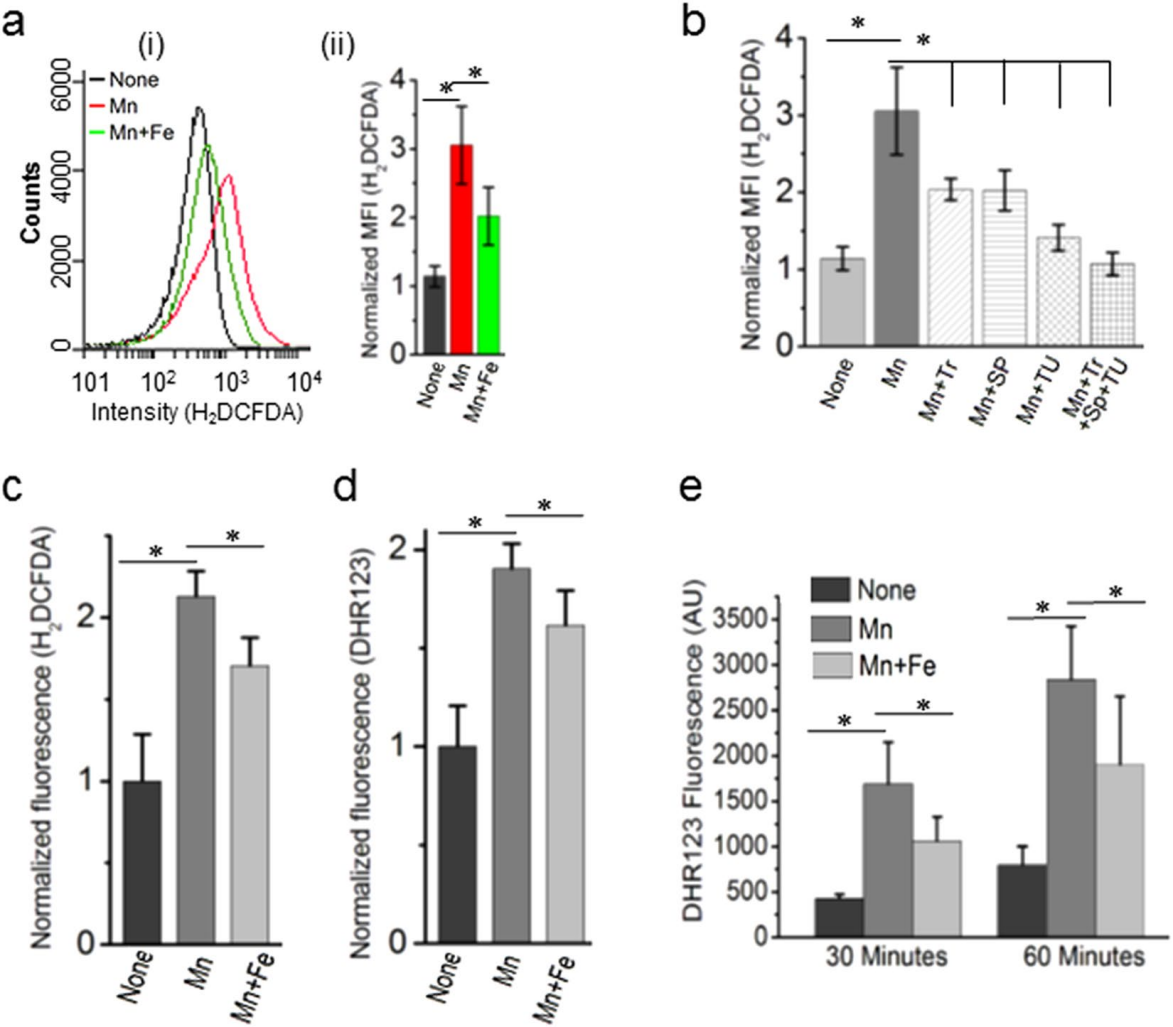
Manganese stress evokes ROS production. (**a)** (i) The FACS data shows the changes in fluorescence intensity of H_2_DCFDA, an indicator dye of oxidative stress, under manganese stress and iron supplementation. Fluorescence intensity is directly proportional to the amount of ROS species in the cell. (ii) Normalized MFI values from three independent experiments are shown by bar diagrams. (**b)** The normalized MFI values of H_2_DCFDA dye under manganese stress and in the presence of ROS quenchers (Tr: tiron, SP: sodium pyruvate, TU: thiourea). (**c**) The H_2_DCFDA fluorescence values were normalized by intracellular dye contents to show that manganese stress evokes ROS production. (**d**) To validate H_﻿2_﻿DCFDA fluorescence results, another dye DHR123 has been used. Increased intracellular dye-normalized fluorescence values of DHR suggests that manganese-fed cells evoke ROS. (**e**) The fluorescence values of DHR123 in the bar diagram indicates that manganese-fed cells produces elevated level of H_2_O_2_ in comparison to the un-fed cells at 30 minutes and 60 minutes time points. Iron-supplemented cells marginally suppressed H_2_O_2_ production. Data are means ± SD (n = 3); *P < 0.001, paired T-test.

Flow cytometry analyses using a cell penetrable ROS sensor dye, 2′,7′-dichlorodihydrofluorescein diacetate (H_2_DCFDA), we show that manganese exposure evoked oxidative stress, while iron supplementation marginally reduced the stress (Fig. 3a). Furthermore, individual ROS quenchers (tiron, sodium pyruvate and thiourea) suppressed oxidative stress to the different extent, indicating that manganese toxicity evoked all the key reactive oxygen species (ROS) (O_2_^**−**^, H_2_O_2_ and ∙OH), respectively (Fig. 3b). Since there are many caveats of using fluorescent sensor probes to measure intracellular ROS^39^, we performed further fluorescence assays using H_2_DCFDA, and another ROS sensor dye, dihydrorhodamine 123 (DHR123). Here, we normalized the fluorescence values by the total cellular dye levels. The data obtained from these experiments show that manganese stress significantly increases the normalized fluorescence of H_2_DCFDA and DHR123. This data confirms that manganese toxicity leads to oxidative stress in Δ*mntP* strain of *E. coli* (Fig. 3c and d). We reason that affected ISC biogenesis allowed adventitious leakage of electrons from flavin mononucleotide (FMN) cofactor of NDH-1^40,41^, generating O_2_^**−**^ that may leads to H_2_O_2_ production in the cells. Therefore, to show H_2_O_2_ production, we incubated the manganese-fed, iron-supplemented and un-fed Δ*mntP* cells in phosphate buffer saline. The extracellular supernatants were collected after 30 and 60 minutes, and 10 µM ferrous ammonium sulphate was added to these. The supernatants from the manganese-fed cells increased DHR123 fluorescence in comparison to the un-fed cells. This data suggests that manganese stress elevates H_2_O_2_ production in Δ*mntP* strain (Fig. 3d).

Many oxidative stress response and redox pathway genes (*sox* *S*, *fur*, *nfs*, *rsx*, *fpr*, *rsx, ahp, gor, grx* and *trx*) were activated in the manganese-fed cells^1^ (u). Upregulated genes that encodes L-cysteine/glutathione exporter (*cyd* *CD*), glutathione, cysteine and methionine synthesizing enzymes (*gsh*A and *cys* genes), and coenzyme-A (Co-A) metabolizing genes (*dfp* and *coa* *A*) (Supplementary Table S3) imply that the cellular reducing environment could be affected under manganese stress^1^. Consistent with this implication, the chronic manganese exposure has been shown to deplete cellular glutathione level in astrocytes and neurons^31^.

### Affected energy metabolism determines manganese toxicity

**Figure 4:**
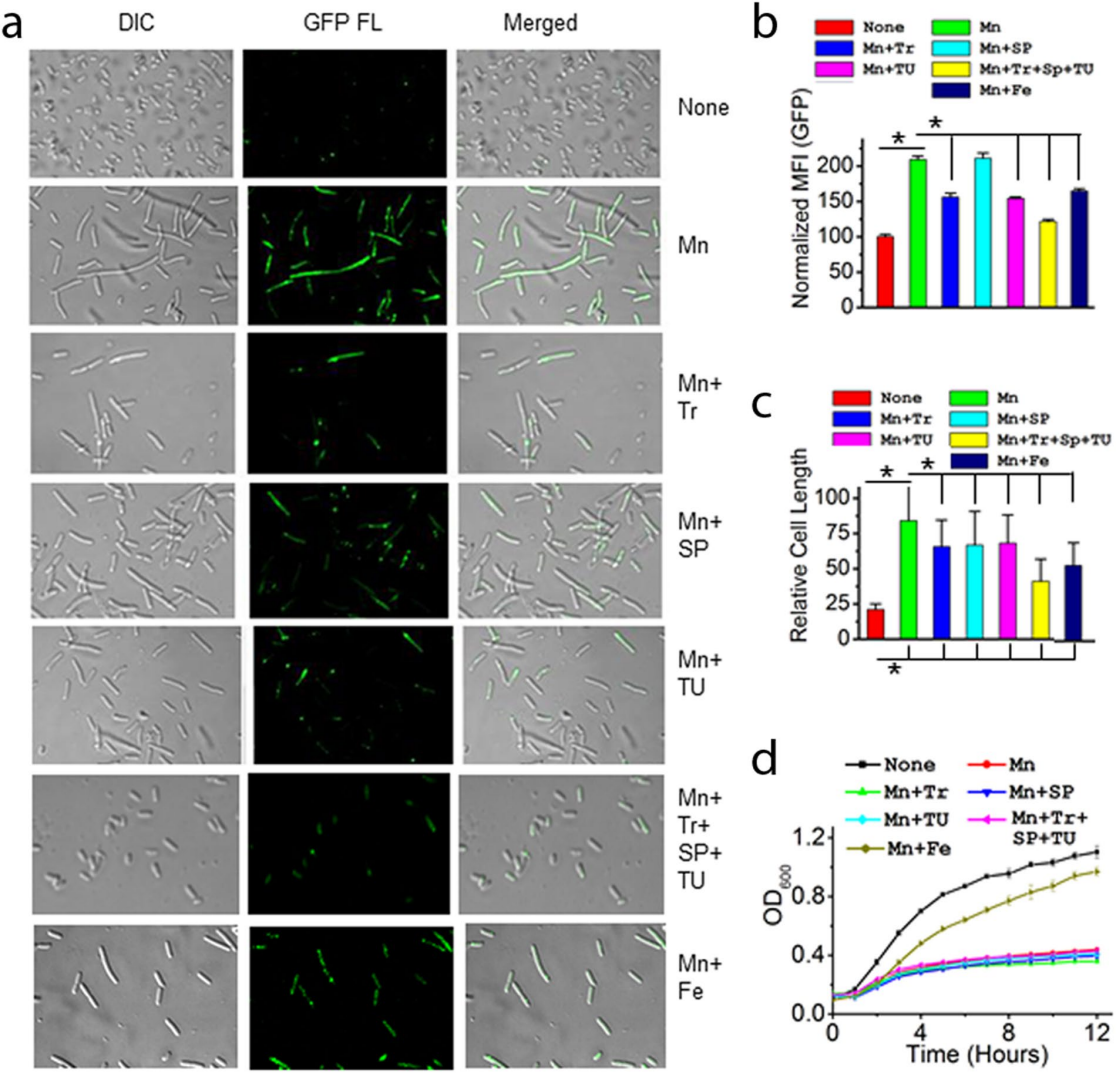
Iron suppresses manganese toxicity, but ROS quenchers failed to do so. **(a)** Confocal microscopy images show that manganese causes cell filamentation and DNA damage. Visibly, iron and ROS quenchers suppressed these phenotypes to some extent. **(b)** Further, GFP fluorescence was quantified by FACS. Normalized MFI values indicate the extents of DNA damages in different conditions. **(c)** The relative cell lengths were measured by confocal images using Image J. software from 75–100 cells. (**d**) The curves show that iron can revive manganese-induced growth arrests, but ROS quenchers failed to do so. Data (B–D) are means ± SD (n = 3); *P < 0.001, paired T-test.

To address whether affected energy metabolism or oxidative damage under manganese stress caused cell toxicity, we checked the cell growth and morphology, ATP level, and DNA damage in the presence of iron supplementation and ROS quenchers (TR, SP and TU). We show that the manganese-fed Δ*mnt*P strain harboring a *rec* *A*:*gfp* fusion^42^ exhibited high GFP fluorescence (Fig. 4a), suggesting that manganese stress induces DNA damage. Besides, increased cell filamentation indicates that manganese stress inhibits cell division (Fig. 4a). Both iron and quenchers were found to suppress DNA damage and cell filamentation to some extent (Fig. 4a–c). However, iron supplementation revived cell growth and ATP production, while the quenchers individually or collectively failed to do so (Figs 4d and 2j). Interestingly, iron failed to suppress oxidative stress to the extent observed in the presence of ROS quenchers (Fig. 3a and b). These data confirm that oxidative stress might play a secondary role in manganese toxicity.

Next, we revisited the pathways to fish out the genes whose differential expressions were reversed by iron supplementation. Iron appeared to resume import of hexose sugars, as indicated by the upregulated expression of the sugar uptake genes (Supplementary Table S4). Interestingly, iron could not repress manganese-activated *isc* and *hem* genes (p). However, iron repressed manganese-induced *nfu* *A* gene expression (p), suggesting that ISC biogenesis was resumed to some extent. Besides iron activated the *suf* genes (Supplementary Table S2) which constitute a parallel system for ISC protein maturation and this could be responsible for reviving ISC protein biogenesis. Partially resumed aconitase and catalase activities by iron supplementation (Fig. 2b and c) confirm that ISC and heme protein biogenesis were restored to some extent. Reduction in cellular pyruvate pool by iron supplementation (Fig. 2g) also indicates that the carbon sources were consumed well by activated TCA cycle. Downregulation of the manganese-activated SoxR/S and OxyR system (Supplementary Table S3) indicates that the revival of electron transport by iron minimized the ROS production (Fig. 2a). All these observations suggests that failure to meet energy demand, but not oxidative stress, is the primary cause of manganese-induced toxicity.

## Discussion

**Figure 5:**
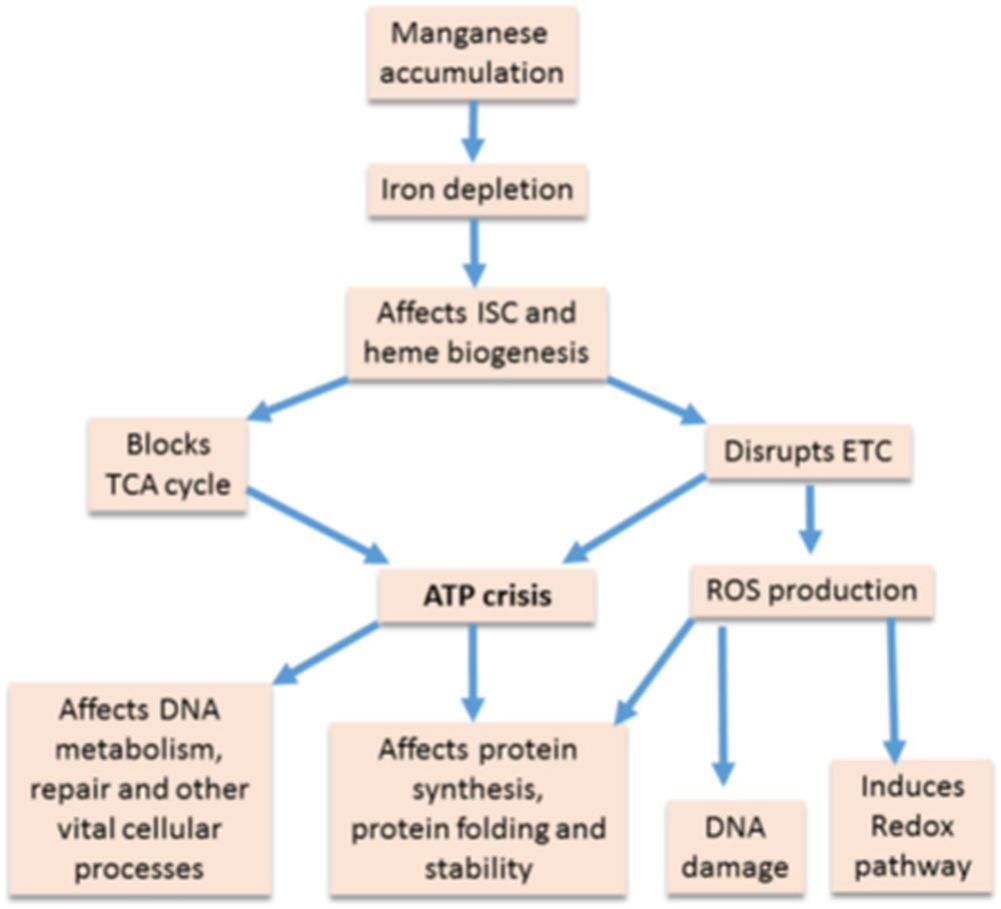
Mechanism of manganese-induced toxicity. Flow-chart showing that manganese accumulation in the cell promotes iron deficiency affecting ISC and heme protein biogenesis. These deficiencies further carbon and energy metabolism to promote ATP crisis in the cells. Flow-chart also shows that affected ISC and heme biogenesis produces ROS. We highlighted that this ATP crisis primarily affects other biological processes, and impair cell growth. On the other hand, ROS could be secondary player to affect some of the cellular proceses.

Our study reveals that manganese stress depletes cellular iron affecting carbon and energy metabolisms (Fig. 2, Supplementary Fig. S4). In addition, improper biogenesis of ETC complexes evokes ROS production (Fig. 2). We address several aspects, as described in the results, to demonstrate that ATP depletion plays a pivotal role to arrest DNA metabolism, cell division, and growth and under manganese-mediated toxicity (Fig. 4). Energy crisis may also play a major role in protein misfolding and degradation by inactivating major cellular chaperones, GroEL/ES and DnaK^1^. In addition, ROS may also act as a secondary factor in protein misfolding and proteolysis (Figs 2 and 3, and Supplementary Fig. S2). We summaries our observations in a flow-chart to show how one pathway affects others to build the observed impact under manganese stress (Fig. 5).

In humans, the chronic exposure of manganese increases its concentration in basal ganglia region of the brain, causing Parkinson’s like neuropsychiatric syndrome^16,17^. Since manganese exposure propagates ROS, many studies propose that the oxidation of the membrane lipids, DNA, amino acids, neurotransmitters, and other biomolecules exert toxicity under manganese stress^18–21^. However, a number of other studies indicate that energy crisis under manganese stress *in vivo* and *in vitro*, including decreases in the activities of mitochondrial enzymes, in membrane potential, and ATP production, could be the cause of neurodegeneration^20–34^. Consistent with a previous literature that manganese exposure affects mitochondrial aconitase activity^43^, we demonstrated that manganese impairs *E. coli* aconitase function (Fig. 2b). Reduced hexose metabolism in the neuronal cells, and inactivation of glycolytic and TCA cycle enzymes also suggest that energy failure could be the cause of manganese toxicity^30,31^. Consistently, analyzing gene expression profile it appears that manganese stress also affects hexose metabolism in *E. coli* (Supplementary Table S4). Similarly, protein misfolding and proteolysis in the manganese-induced neurodegeneration^44^ can be equated with the proteolysis in the manganese-fed *E. coli* strain (Supplementary Fig. S1, Supplementary Table S2), though mechanism of activation of proteolytic pathways are different in *E. coli* and mammals. Such protein misfolding and degradation can be the result of energy crisis or oxidative stress. Addressing both the possibilities, our study determines that the energy crisis is the primary cause of manganese toxicity in *E. coli*. We must mention here that the exogenous oxidative stress has its ability to induce iron scarcity and manganese abundance, affecting ATP production^5,45,46^. Thus, manganese and iron homeostasis, ATP production and oxidative stress to counterbalance each other under changing environmental milieu. Interestingly, manganese-fed *E. coli* cells elevated relative cellular pH, and pyruvate (Fig. 2k and g). Here, pyruvate could act as a quencher of H_2_O_2_ species under such basic cellular pH^47,48^.

Microarray data indicates that the rewiring of gene expression network in the manganese-fed cells also facilitated aerobic ATP synthesis. First, a complete repression of the genes and operons encoding flagellar biosynthesis and motility proteins (*flg*, *fli*, *mot*, *etc*.) attracts particular attention (Supplementary Table S3). Such rewiring of gene expression implies that cells prioritize ATP production to utilize PMF economically, sacrificing the PMF-driven flagellar movements^1^. Second, for effective utilization of limited iron cofactors for ATP production, manganese stress activated ATP synthase (*atp*), and aerobic respiratory genes (the *ndh*, *nuo* and *sdh*, *ubi*) (Supplementary Table S3). Third, activation of *cyd* *CD* genes (Supplementary Table S3), which encodes glutathione/L-cysteine exporter protein^1^, indicates that the manganese-fed cells prepares itself to maintain periplasmic redox state, helping heme ligation during cytochrome-*bd* assembly. On the contrary, downregulation of *ccm* genes (Supplementary Table S2), which encodes heme-assembling protein complex required for cytochrome-*c* biogenesis^1^, indicates that manganese toxicity may block the utilization of heme for anerobic respiration. Furthermore, since the function of many other anaerobic respiratory proteins depends heavily on ISC, heme and Moco prosthetic groups^1^, manganese stress repressed the genes in the anaerobic (nitrate, nitrite, xanthine, DMSO and fumarate) respiratory pathways (Supplementary Table S3), favoring iron utilization for aerobic respiration. In addition, downregulation of nitrogen respiration genes (Supplementary Table S3) could inhibit the production of nitric oxide (NO∙) and peroxynitrite radical (ONOO∙), which are highly damaging to glycolytic and ETC enzymes^49,50^. Therefore, cessation of NO∙ synthesis under manganese stress may prevent further damage to the impaired ETC. Third, manganese toxicity repressed formate-metabolizing genes (*tdc*, *hyb*, *hyd*, *hyc, hyf*, *frd* etc.) (Supplementary Table S4). Such repression also curtailed unwarranted utilization of ISC and heme by formate-metabolizing enzymes. In addition, these enzymes require nickel-iron (Ni-Fe) cluster for their functions^1^. Our data show that the repression of the nickel (Ni) transporter gene (*nik*) (Supplementary Table S4) reduced Ni level (Fig. 2a) in the manganese-fed cells. This low nickel level would be a limiting factor for Ni-Fe cofactor production.

Activation of the peptidoglycan and envelope component (fatty acids, lipids, and biotin) biosynthesis genes (Supplementary Table S4) raises a question whether poor envelope biogenesis in the manganese-fed cells was also affected. We observed that the viability of manganese-fed cells was reduced considerably compared to the unfed cells upon SDS/EDTA treatment (Supplementary Fig. S6). Treating the cells with lysozyme or ultrasound also reduced the survivability of manganese-treated cells (Supplementary Fig. S6), suggesting that overall membrane biogenesis is profoundly affected. Supplementation of iron improved the survivability, which was almost comparable to the survivability of unfed cells (Supplementary Fig. S6), suggesting that manganese stress damages cell envelope by impairing iron metabolism. Therefore, apart from the scarcity of ISC, heme, NADH and ubiquinone, the damaged envelope under manganese stress could also failed to maintain a robust proton gradient across the membrane, lowering ATP synthesis.

## Materials and Methods

### Bacterial strains, phages, growth conditions

Δ*mnt*P and other knockout strains of *E. coli* used were collected from KEIO library^29^. Double knockout mutants were generated as described^51^. An automated BioscreenC growth analyzer (Oy growth curves Ab Ltd.) was used to generate growth curves. Overnight culture of Δ*mnt*P strain was diluted 100 times in fresh LB medium and grown in the presence and absence of 1 mM MnCl_2_, or 1 mM MnCl_2_ + 0.5 mM FeCl_3_) for 2 hours followed by microarray, real-time PCR and proteomics analysis. ODs of the Δ*mnt*P cultures were kept less than 0.4 for microarray, 2D proteomics and all biochemical assays. The *E. coli* cells harvest point has been shown in the Supplemetary Fig. S7.

### Microarray and proteomics experiments

The mRNA isolation, quality control, sample labeling, single color hybridizations with E coli_Gxp_8 × 15 K array from Agilent technologies, scanning and feature extraction was done at Genotypic Pvt. Ltd., Bangalore. The raw data thus obtained was analyzed by in-house coded R Scripts (https://cran.r-project.org/). For 2D experiments, the proteins were extracted from the manganese-fed and unfed cells by methanol/chloroform method^52^. The 1^st^ dimensions of 2D gels were performed using BioRad pI strips (17 cm, 3–10 L). The 2^nd^ dimension SDS-PAGE was done using 15% resolving gels.

### ICP-MS to determine intracellular metal level

Metal contents were determined by ICP-MS facility provided by Punjab Biotechnology Incubator, Mohali, India. The metal concentration in the cell was determined by normalizing them to a total intracellular protein concentration of 300 mg/ml, as described previously^5^.

### Measurement of survivability

Δ*mnt*P cells were grown in LB broth in the presence or absence of 1 mM MnCl_2_ up to 2 hours. One portion of the cell cultures was perturbed with SDS/EDTA (0.05% SDS/0.8 mM EDTA) for 10 minutes^53^. Next, the cells were serially diluted to spread them on LB agar surface. Colonies were counted next day and compared with the numbers of colonies appeared from unperturbed cells to calculate the survivability. Similarly, cells were treated with lysozyme (10 μg/ml) for 10 minutes, or sonication (10 seconds pulse) and the survivability calculated.

### Biochemical assays

Relative ATP measurement was done using ATP Bioluminescence Assay Kit CLS II, Roche. ROS levels were detected by H_2_DCFDA and DHR123 fluorescent dyes (Thermo-Fisher). We used DHR123 probe along with 10 µM ferrous ammonium sulfate to detect extracellular H_2_O_2_. 10 mM of each ROS quenchers (tiron, thiourea and sodium pyruvate) were used. The fluorometric intracellular pH assay kit (Sigma) was used to monitor intracellular pH change. Colorimetric assay using methylene blue has been performed in a tightly sealed microfuge tube containing growing *E. coli* cells in the aerated LB medium. Reazurine dye has been added in the aerobically growing bacterial culture to see the color change. Colorimetric assay kits (Abcam) were used to detect relative levels of pyruvate, NAD, NADH in the manganese-fed, (manganese + iron)-fed and unfed cells. Aconitase, catalase, NDH-1, SDH, and glutamate synthase (GS) assays were performed, as described^54–57^. Relative ubiquinone level in the cells was determined as described by Chehade *et al*.^58^.

### Confocal microscopy and flow cytometry experiments

Imaging was done using Nikon confocal microscope using 488 laser. Flow cytometry experiments were performed using BD FACS acuri instrument for 0.1 million cells using FL1 laser. The relative cell lengths were measured by confocal images using Image J. software from 75–100 cells.

### Data Availability

All data generated or analyzed during this study are included in this published article, and its Supplementary Information files). The detailed microarray array raw and normalized data were deposited in the GEO database with accession number GSE88906.

## Electronic supplementary material

Supplementary materials
