# Supplementary material for: Affected energy metabolism under manganese stress governs cellular toxicity
Source: Sci Rep. 2017 Sep 19;7:11645. doi: 10.1038/s41598-017-12004-3 (PMC5605510; doi:10.1038/s41598-017-12004-3)

Supplementary material

Affected energy metabolism under manganese stress governs cellular toxicity

Gursharan Kaur[1, 2, 3], Vineet Kumar[1, 3], Amit Arora[1], Ajay Tomar[1], Ashish[1], Runa Sur[2], Dipak Dutta[1, 4]

[1]CSIR-Institute of Microbial Technology, Sector 39-A, Chandigarh 160036, India

[2]Department of Biophysics, Molecular Biology & Bioinformatics, Calcutta University, Kolkata, India

[3]Equal contributions

[4]Corresponding author

Contact: dutta@imtech.res.in

Materials and Methods

Growth condition for microarray analysis

Overnight culture of Δ*mnt*P strain was diluted to 1:100 in Luria broth and grown in the presence or absence of $MnCl_2$ (1mM) and $MnCl_2$ supplemented with 0.5mM $FeCl_3$ for 2 hours at 37°C. 2.5 ml stop solution (5% phenol in ethanol) was added in 25 ml of growing culture for 10 minutes to prevent the RNA degradation and the cell pellets were collected by centrifuging at 5000 rpm for 10 minutes at 4°C.

RNA Extraction and RNA Quality Control for Microarray

E. coli pellet was re-suspended in 300ul of 5mg/ml lysozyme and incubated at Room Temperature (RT) for 30 min. Isolation of RNA from *E. coli* was carried out by using Qiagen RNeasy mini kit (Cat # 74106) as per manufacturer's guidelines including DNase treatment step. The purity of the RNA was assessed by using the Nanodrop Spectrophotometer (Thermo Scientific; ND-1000) and the integrity of the RNA was analyzed on the Bioanalyzer (Agilent; 2100). We considered RNA to be of good quality based on the 260/280 values (Nanodrop), rRNA 28S/18S ratios and RNA integrity number (RIN) (Bioanalyzer).

Microarray Labeling

The sample labeling was performed using Quick-Amp Labeling Kit, One Color (Agilent Technologies, Part Number: 5190-0442). 500ng of each samples were denatured along with WT primer with a T7 polymerase promoter. The cDNA master mix was added to the denatured RNA sample and incubated at 40°C for 2 hours for double stranded cDNA synthesis. Synthesized double stranded cDNA were used as template for cRNA generation. cRNA was generated by *in vitro* transcription and the Cyanine-3-CTP (Cy3- CTP) dye

incorporated during this step and incubated at 40°C for 2:30 hours. The Cy3-CTP labeled cRNA sample was purified using Qiagen RNeasy column (Qiagen, Cat # 74106). The concentration of cRNA and dye incorporation was determined using Nanodrop-1000.

Microarray hybridization and scanning

About 4 microgram of labeled Cy-3-CTP cRNAs were fragmented at 60°C for 30 minutes and the reaction was stopped by adding 2X GE HI-RPM hybridization buffer (Agilent Technologies, In situ Hybridization kit, Part Number5190-0404). The hybridization was carried out in Agilent's Surehyb Chambers at 65°C for 16 hours. The hybridized slides were washed using Gene Expression Wash Buffer1 (Agilent Technologies, Part Number 5188-5325) and Gene Expression Wash Buffer 2 (Agilent Technologies, Part Number 5188-5326) and was scanned using Agilent Scanner (Agilent Technologies, Part Number G2600D). Data extraction from the images was done using Feature Extraction Software Version 11.5.1.1 of Agilent.

Microarray data analysis

Microarray data analysis was undertaken by in-house coded R Scripts (https://cran.r-project.org/). Processing of raw data into expression profiles was achieved by utilizing the packages limma and affy. Heatmaps were generated by the package ggplot2. Probe intensities were converted into expression measures by standard procedures. Briefly- the design-sets depicting the "control/test" arrays were carefully generated by reading the raw data from MA image files. Background correction was done by the method "normexp". This data was quantile normalized (between arrays depending on the design set) and within array replicates were averaged. Model fitting and empirical

Bayes statistical analysis was done using lmFit() and eBayes() methods. This analysis was repeated independently for only protein coding genes and intergenic regions.

Real Time PCR

Bacterial mRNAs were isolated by Trizol reagent method using Qiagen Kit. The RNA concentration was determined by a Nano-drop spectrophotometer. 250ng-500ng of RNA samples and one-step reaction mixture (Invitrogen) were used for RT-qPCR. At least three independent experiments were conducted for each primer pairs and fold changes were determined from C_t value differences between manganese-fed and unfed samples.

2D gel eletrophoresis

Mn-fed (1mM) or -unfed cells were resuspended in lysis buffer (20 mM Tris, pH 8, 200 mM NaCl, 0.3% SDS, Complete™ protease inhibitor cocktail (Roche), DNase I and RNaseA) and lysed by sonication. Proteins were extracted by methanol/chloroform method as described [30]. Dried protein samples were allowed to dissolve overnight in 7 M urea, 2 M thiourea, 4% w/v CHAPS. Total protein was estimated by Bradford reagent (Biorad). 1st dimension of 2D gel was performed using BioRad pI strips (17cm, 3-10L). Equal amounts of proteins (1mg/strip) were mixed with Bio-Rad IEF buffer along with ampholytes 3-10 and soaked to the pI strips under an overlay of mineral oil for 14 hours. IEF was performed in Protein IEF Cell (BioRad) for 40,000 Volt-Hour using a preset program. The 2D strips were removed from the IEF cell. Mineral oil was removed by soaking it with wet Whatman filter paper and incubated with SDS PAGE gel loading dye solution for 30 minutes. 10% and 14% resolving SDS-PAGE gels were prepared leaving 2 centimeter gap on the top using a BioRad vertical electrophoresis system. The stained pH strip was placed horizontally in the gap on the top of the gel and between two glass plates.

Low melting agarose was poured to remove any bubble between polyacrylamide and pH strip. The 2nd dimension electrophoresis was performed at 100 volts till the dye front reaches at the bottom. 2D gels were stained with coomassie brilliant blue R (CBB-R) and documented, as represented in the Fig. 1B. The protein spots which are labeled in the gel (Fig. 1B) were excised and trypsinized accordingly[59].

In-gel trypsinization of 2D spots

Each spot was excised with sterile blade, collected in a microfuge tube and chopped into smaller pieces. All samples (spots) were washed with sterile water and resuspended in 200-500µl destaining solution (100mM ammonium bicarbonate and acetonitrile (ACN) mix (1:1)), for 30 minutes at room temperature. Tubes were centrifuged for 5000rpm for 1 minute and supernatant was discarded. The gel pieces were resuspended in 100-200µl ACN for another 30 minutes at room temparature. The centrifugation step was repeated, liquid discarded and gel pieces dried in speed vacuum centrifuge for 30 min at 30°C. The protein samples were dissolved in 30-50µl of Trypsin-HCl buffer (13ng/µl trypsin) (Promega) prepared in 50mM ammonium bicarbonate and 1mM HCl and kept for 2 hours at 37°C. The tubes were centrifuged briefly and 5-10 µl of sample elutes were taken for MS/MS.

Tandem MS/MS analysis

All experiments of MS and tandem MS were performed on an AB SCIEX MALDI TOF/TOF 5800 mass spectrometer with delayed extraction time 450 nanosecond to obtain maximum resolution. Matrix was prepared from α-cyano-4-hydroxyl cinnamic acid in 50% acetonitrile plus 0.1% TFA. Trypsinized samples and matrix were mixed in 1:1 ratio. MS data was acquired at a laser repetition rate of 400Hz with total 1600 laser shots/spectrum. Tandem MS data was acquired at 1000Hz in 1KV MS/MS mode with 2000 laser

shots/spectrum. Experiments were performed without DynamicEXIT algorithm and interpretation method was set for 40 strong precursors. TOF/TOF Explorer Series software was used for data acquisition and processing. The MS/MS files were analyzed by Mascot program (http://www.matrixscience.com/) to identify the proteins.

ICP-MS to determine intracellular metal levels

To determine the cellular metal contents, we grew the Δ*mnt*P strain of *E. coli* in the presence or absence of manganese. After three hours, the cell pellets were harvested and washed twice in milliQ water containing 1mM EDTA to remove cell surface bound metal ions. The cell pellets were dissolved in 2 ml of clean double distilled water and protein content was measured from a portion of the suspension. The remaining amount of cell suspensions were digested with concentrated nitric acid and 30% H_2O_2. The digested samples were filtered to get rid of any particles and metal contents were determined by ICP-MS facility provided by Punjab Biotechnology Incubator, Mohali, India. The metal concentration in the cell was determined by normalizing them to a total intracellular protein concentration of 300mg/ml, as described previously[5].

Aggregated protein isolation from the cell extract

Cell pellets from 50 ml Δ*mnt*P strain (Mn-fed or unfed) were harvested, suspended in 250 μl of buffer A (10mM potassium phosphate buffer, pH 6.5, 1mM EDTA, 20% sucrose, 1mg/ml lysozyme) with protease inhibitor cocktail (Roche), and incubated for 30 minutes. 500 μl of buffer B (10mM Potassium phosphate buffer, pH 6.5, 1mM EDTA) was added thoroughly; samples were flash frozen in liquid nitrogen, and sonicated briefly. The cell lysates were centrifuged at 2000g for 10 minutes to remove cell debris and intact cells. The protein concentrations in the supernatants were estimated by Bradford reagent

(BioRad). 200 µg of total proteins from each samples were taken out and mixed with the loading dye to visualize in the SDS-PAGE. The lysed cells containing equal amount of proteins were centrifuged at 15000g for 45 minutes at 4°C. The pellets with inclusion bodies or aggregated proteins were resuspended in 500 µl buffer B and centrifuged again at 15000g for 45 minutes at 4°C. Pellets were collected and washed with 10% NP-40 and centrifuged at 15000g to remove membrane components. Pellets were finally resuspended in 30 µl buffer B for SDS-PAGE analysis.

Western Blotting

Δ*mnt*P cells were grown to log phase in the presence and absence of 1mM manganese for 2 hours. Subsequently, 20µg/ml chloramphenicol and 10 µg/ml tetracycline were added to the culture and cells were collected at 0 15, 30 min time points and washed with saline before sonication in lysis buffer (20 mM Tris, pH 8, 200 mM NaCl and protease inhibitor cocktail buffer). Protein concentration was estimated by Bradford and equal amount of lysate were loaded for control and treated samples in 12% SDS-PAGE. Semi-dry transfer system was used to transfer proteins onto nitrocellulose membrane for 1hr at constant volt (10V). The blot was blocked in 5% skimmed milk in TBST (Tris-NaCl buffer with 0.01% Tween-20) buffer, followed by incubation with primary antibody (rabbit anti-TF) (Sigma) and secondary antibody (Goat anti-rabbit HRP conjugate) for 1 hour at room temperature followed by three washes with TBST after each incubation. The blot was developed with WesternBright^{TM} ECL western blotting detection kit (Advansta) and observed in BioRad Imager. Scion image software was used to measure anti-TF band intensities and normalizations were done against band intensity at 0 minutes.

Aconitase activity assay

Cell pellets were resuspended in 1ml of aconitase lysis buffer (0.1mM Tris-HCl, pH 8.0, 0.1M KCl, 1mM PMSF, and 0.6ug/ul lysozyme), and lysed by 5-6 cycles of freeze & thaw in liquid nitrogen. The lysate was centrifuged at 14000 rpm for 10 min at 4°C. Aconitase assay was performed by adding 100ug of protein to 200ul of 1X aconitase assay buffer (0.6mM $MnCl_2$, 25mM sodium citrate, 0.25mm NADP+, 50mM Tris-HCl, pH 7.6). The OD was recorded at 340nm. Absorbance is directly proportional to the cellular aconitase activity. Relative aconitase activity was calculated against per milligrams of cellular proteins.

Catalase activity assay

Catalase assay was performed by foam forming ability of the Δ*mnt*P strain carrying mutated plasmids in the presence of H_2O_2, as described [33]. Briefly, manganese-fed and unfed cells pellets were harvested from 10 ml log phase cultures and resuspended in 100μl normal saline. 25 μl was used to estimate the proteins and remaining portions were placed in the narrow glass test tubes. Addition of concentrated H_2O_2 produced foam in the tube. The heights of the foam correspond to the activity of the cellular catalases. Relative catalase activity was calculated against per milligrams of cellular proteins.

NDH-1 and SDH activity assays

NDH-1 and SDH assays were performed as mentioned[57]. Briefly, untreated, manganese-fed and iron-supplemented cells were collected by centrifugation. The cell pellets were suspended in 50 mM MES buffer (pH 6.0) with 10% glycerol and lysozyme 50µg/ml, and disrupted by sonication. Cell debris were separated by low speed centrigugation. One-halves of the cell extracts were directly used to test NDH-1 activity.

The other-halves of the cell extracts were used to prepare membrane fraction centrifuging at 45,000 *g* for 2 hours. For NDH-1 activity assay, a specific substrate, deamino-NADH has been used. The oxidation of deamino-NADH was monitored using a spectrophotometer at 340 nm (εNADH = 6.22 mM^{-1}. cm^{-1}), in a reaction mixture containing 50 mM MES (pH 6.0), 10 % glycerol, 200 μM deamino-NADH. Relative NDH-1 activities were determined against per milligrams of cellular proteins. For SDH activity assay, the membrane faction was suspended in the same buffer, and dichlorophenol indophenol (DCPIP) reduction was monitored in 50 mM Tris-HCl (pH 7.5), 4 mM succinate, 1 mM KCN for 30 min at 30°C. The activity was determined spectrophotometrically by following the phenazine ethosulfate (PES)-coupled reduction of DCPIP at 600 nm (εNADH = 20.7 mM^{-1}. cm^{-1}), in a reaction mixture containing 50 mM Tris-HCl (pH 7.5), 4 mM succinate, 1 mM KCN, 400 μM PES and 50 μM DCPIP. Relative SDH activities were determined against per milligrams of cellular proteins.

Glutamate synthase (GS) assay

GS assays were performed as mentioned[58]. Briefly, growing cells were collected by centrifuging at 12000 *g* for 10 min. The cell pellets were sonicated in the buffer containing 0·1 M KCl and 0·5% (v/v) 2-mercaptoethanol, adjusted to pH 7·6, and centrifuged to remove cell drbris. GS activity was determined using a spectrophotometer by following the glutamine-dependent oxidation of NADPH at 340 nm. The reaction mixture contained 50 mM HEPES buffer pH 8·5, 1% (v/v) 2-mercaptoethanol, 3·65 mM glutamine, 3 mM 2-oxoglutarate, 0·2 mM NADPH and 0·1 ml cell-free extract in a final volume of 1 ml. Relative GS activities were normalized per milligram of cellular proteins.

Estimation of intracellular NAD and NADH

Abcam colorimetric assay kit was used to estimate NAD/NADH levels. 10 ml log phase culture of *E. coli* was grown in the presence or absence of 1mM MnCl2 or MnCl2+0.5mM FeCl2 for 2 hours and washed with cold PBS. Extraction of NADH/NAD were done by adding 400 µL of extraction Buffer followed by 4 freeze-thaw cycles on liquid nitrogen and by sonicating briefly. The lysates were centrifuge for 5 minutes at 4°C at 15000 rpm. The supernatants were collected and passed through 10kD spin column to remove NAD/NADH decomposing before performing the assay. Half of the filtrates were used directly with assay reagents to measure total NAD levels (NADt=NAD+NADH). Remaining halves were incubated at 60°C for 30 minutes to decompose NAD, and assay was performed to measure NADH levels. Relative NAD or NADH levels were calculated against per milligrams of cellular proteins.

Determination of intracellular ATP level

Relative ATP measurement was done using ATP Bioluminescence Assay Kit CLS II, Roche. Briefly, cell pellets were collected and washed in 1X PBS. The cell pellets were resuspended in pre-boiled ATP extraction buffer (100 mM Tris, pH 7.75 and 4mM EDTA, pH 8.0) and one small portion was taken out to measure protein concentration. The remaining portions were incubated for 2 minutes at 100°C. The samples were centrifuged for 5 minutes at 1000g and the supernatant was transferred to the fresh microfuge tubes. 50µl of sample and 50 µl of luciferase reagent were added in Black 96 well microplate and luminescence were recorded using a luminometer. The relative light unit (RLU) values were recorded. Standard curve was generated using known ATP concentrations. Finally, ATP levels were normalized against per milligrams of proteins.

Probing the intracellular and extracellular reactive oxygen species (ROS)

ROS species were detected by H_2DCFDA and DHR123 fluorescent dyes (Thermofisher). To measure the intracellular ROS using flow cytometry, approximately equal number of cells were taken (different conditions) and pellet was done. Cell pellet was washed with 1X PBS and distributed in two parts. In one part 1X PBS was added and in another part equal volume of 10µM H_2DCFDA dye was added. Staining was done for one hour and the data was acquired using FACS acuri (BD) at FL1 laser for 0.1 million cells. The background fluorescence values of the cells were subtracted and the data were normalized by side scattering values. We also used a fluorimeter to measure total cellular fluorescence under different conditions. For this, equal mass of the cell pellets from manganese-fed, iron-supplemented and unfed control were collected. The cells were incubated with or without 10µM H_2DCFDA or 200nM DHR123 probes. The cell pellets were collected by centrigugation, and were washed twice with the ice-cold PBS buffer. The resuspended cell pellets were divided into two equal halves. One-half was used to measure the fluorescence values originated by the internal oxidation of dye by cellular •OH species using a standard fluorimeter. The other half was incubated with 20mM H_2O_2, expecting that the H_2O_2 to enter the cells and produce enough •OH species to oxidize all dye molecules present in the cells. The background fluorescence of untreated cell suspensions were subtracted. We normalize the fluorescent values by the total fluorescence of the cellular dyes observed under H_2O_2 treatments. To measure extracellularly secreted H_2O_2, we used DHR123 probe in combination of 1µM ferrous ammonium sulfate. For this, equal mass of treated and untreated cell pellets were incubated in PBS buffer for 30 minutes and 60 minutes. Cellular supernatants were collected and ferrous ammonium sulphate was

added. Then DHR123 probe was added and fluorescent values were recorded after 10 minutes.

Measuring relative intracellular pH changes

To measure the relative intracellular pH changes, fluorometric intracellular pH assay kit (Sigma-MAK150) was used. The protocol was little modified because kit is standardized for eukaryotic cells. Around 0.1 million control, manganese-fed and iron supplemented cells were harvested and washed in 1X PBS. After washing the cells were suspended in HHBS buffer supplied with Kit and distributed in two halves. In one half HHBS buffer was added and in another half dye loading solution was added (composition as described in kit). Staining was done for one hour and immediately the data was acquired using FACS acuri (BD) for one lakh cells using FL1 laser.

Estimation of intracellular pyruvate level

We used pyruvate colorimetric assay kit (Abcam). Cells were resuspended in pyruvate assay buffer and sonicated in ice for 2-4 min. Portions of lysates were taken out to measure the protein concentrations by Bradford method. The lysate was de-proteinised as recommended in the protocol. The samples were centrifuged at 13000 rpm for 15 min in cold before the assay was performed. 50μl of lysate was used for reaction with probe and enzyme mix provided in the kit following the mentioned protocol and blanks were set with pyruvate buffer as suggested. The OD was taken at 570 nm using a spectrophotometer. Standard curve was prepared using known concentration of pyruvates. Pyruvate level was normalized against per milligrams of cellular proteins.

DNA damage studies by Confocal and FACS

To study DNA damage in presence of manganese and array of other chemicals (ROS scavengers and Spermidine) in combination with manganese confocal microscopy and flow cytometery was used. The cells were grown for 4 hours in the presence of 1 mM $MnCl_2$ and pelleted. Pellet was washed with 1X PBS and the cells were fixed in 4% formaldehyde at 37°C for 10 minutes. The cells were again pelleted, washed and finally dissolved in 1X PBS. 10µl sample was used to prepare slides and rest of the sample was used for flow cytometry study. FACS data was acquired using FACS acuri (BD) for 0.1 million cells using FL1 laser and imaging was done using Nikon confocal microscope using 488 laser. The relative cell lengths were measured by confocal images using Image J. software from 75-100 cells.

Measurement of survivability

Δ*mnt*P cells were grown in LB broth in the presence or absence of 1 mM $MnCl_2$ up to 2 hours. One portion of the cell cultures was perturbed with SDS/EDTA (0.05% SDS/ 0.8mM EDTA) for 10 minutes (32). Next, the cells were serially diluted to spread them on LB agar surface. Colonies were counted next day and compared with the numbers of colonies appeared from unperturbed cells to calculate the survivability. Similarly, cells were treated with lysozyme (10µg/ml) for 10 minutes, or sonication (10 seconds pulse) and the survivability calculated.

Supplementary Text

Manganese promotes unfolding stress, elevating protein aggregation and degradation

Growing *E. coli* Δ*mnt*P cells in LB broth containing 1 mM supplemental $MnCl_2$ for 2 hour results in poorly adhesive white cell pellet, which was visibly different from the firmly adhesive yellowish cell pellet harvested from the unsupplemented culture, led us to believe that that the intracellular inclusion body could be formed. Fractionation of the soluble and aggregated proteins revealed that a substantial number of the cellular proteins were aggregated (Supplementary Fig. S2a). In corroboration, the microarray data also interrogate protein misfolding. Activated chaperones and unfolding stress relievers (*tig*, *dna*KJ, *grp*E, *gro*SL, *ibp*AB, *hsl*O, *hsl*R, *etc*.), Peptidyl-prolyl *cis-trans* isomerases (*fkp*A, *ppi*A, *sur*A etc.), and protein secretion systems genes (*sec*AB, *tat*A *etc*.) reflects that manganese promotes unfolding stress (Supplementary Table S1). Several chemical chaperones (viz. glycerol, alanine, proline, glycine betaine, pantethine and arginine) transporting and synthesizing genes were upregulated[60, 61] (Supplementary Table S1). Since ribosome exhibits chaperone function[62, 63], an elevated expression of ribosomal genes (Supplementary Table S1) might also be linked to the protein folding. Repressed profile of fimbrial genes (Supplementary Table S1) suggests why manganese stress reduces the adhesive properties of the cells.

The protein aggregation activated almost all cytoplasmic protease coding genes (*lon*, *hsl*V, *pep*Q etc.) (Supplementary Table S1), indicating that unfolding stress stimulates the protein degradation. Interestingly, while periplasmic chaperones genes (*sur*A and *skp*) were upregulated, *omp*T protease gene was repressed (Supplementary Table S1). Out of

these, upregulation of PepQ peptidase under manganese stress could only be detected in the 2D gel (Figs. 1b and 1c). Some protein spots including outer membrane proteins (OMP) (OmpA, SodA, and IscS *etc.*) were found to be downregulated, though they were upregulated or not differentially expressed at mRNA level under manganese stress (Figs. 1b and 1c). We reason that activation of proteolysis could limit the cellular concentration of these proteins.

To test the activated protein degradation, we focused on the TF chaperone levels in the cells. We selected TF because of three reasons. First, *tig* mRNA level was increased about 4-fold under manganese stress, but protein level was indifferent (Figs. 1b and 1c). Second, TF plays a central role in protecting nascent OMPs until they are secreted to the periplasm[53, 64]. Third, anti-TF antibody was available in the market to detect its cellular level. For this, we blocked the translation of mRNAs in the growing cells for different time points (Supplementary Fig. S2b) by chloramphenicol and tetracycline. TF level was found to be declined rapidly under manganese stress in the absence of translation, suggesting that TF chaperone itself was highly unstable under manganese-induced unfolding and proteolytic stress (Supplementary Fig. S2b). Furthermore, the Δ*mnt*PΔ*tig* mutant showed a similar growth profile of Δ*mnt*P strain (Supplementary Fig. S2c), suggesting that manganese-induced unfolding stress might fully inactivate the TF. Thus, inactivated TF apparently could not protect OMPs from the cytoplasmic proteases under manganese stress.

We further addressed the contributions of Lon protease and DnaK chaperone, which were upregulated at the transcription level, under manganese stress (Supplementary Table S1). Both the Δ*mnt*PΔ*lon* and Δ*mnt*PΔ*dna*K strains exhibited slower growth in

comparison to Δ*mnt*P strain under manganese stress (Supplementary Figs. S2d and S2e). The growth defect in the absence of DnaK chaperone is important in this regard since it is known to compensate TF function[65]. These experiments suggest that proteases and chaperone systems played crucial roles under manganese stress. The protein aggregation under manganese stress may be attributed to the many factors. Instability of TF chaperone and reduced chaperone function, endogenous ROS, exhausted ATP levels (Supplementary Fig. S2; Fig. 2, Fig. 3), and even infidelity of polymerases[66-68] in the manganese-stressed cell together could contribute in protein aggregation.

References

59 Shevchenko, A., Tomas, H., Havlis, J., Olsen, J. V. & Mann, M. In-gel digestion for mass spectrometric characterization of proteins and proteomes. Nature protocols 1, 2856-2860 (2006).

60 Welch, W. J. & Brown, C. R. Influence of molecular and chemical chaperones on protein folding. Cell Stress Chaperones 1, 109-115 (1996).

61 Clark, J. I. & Huang, Q. L. Modulation of the chaperone-like activity of bovine alpha-crystallin. Proc Natl Acad Sci U S A 93, 15185-15189 (1996).

62 Das, D. et al. Role of the ribosome in protein folding. Biotechnol J 3, 999-1009, doi:10.1002/biot.200800098 (2008).

63 Kaiser, C. M., Goldman, D. H., Chodera, J. D., Tinoco, I. & Bustamante, C. The ribosome modulates nascent protein folding. Science 334, 1723-1727, doi:10.1126/science.1209740 (2011).

64 Crooke, E., Brundage, L., Rice, M. & Wickner, W. ProOmpA spontaneously folds in a membrane assembly competent state which trigger factor stabilizes. EMBO J 7, 1831-1835 (1988).

65 Deuerling, E., Schulze-Specking, A., Tomoyasu, T., Mogk, A. & Bukau, B. Trigger factor and DnaK cooperate in folding of newly synthesized proteins. Nature 400, 693-696, doi:10.1038/23301 (1999).

66 El-Deiry, W. S., Downey, K. M. & So, A. G. Molecular mechanisms of manganese mutagenesis. Proc Natl Acad Sci U S A 81, 7378-7382 (1984).

67 Imashimizu, M., Tanaka, K. & Shimamoto, N. Comparative Study of Cyanobacterial and E. coli RNA Polymerases: Misincorporation, Abortive Transcription, and Dependence on Divalent Cations. Genet Res Int 2011, 572689, doi:10.4061/2011/572689 (2011).

68 Zakour, R. A., Kunkel, T. A. & Loeb, L.A. Metal-induced infidelity of DNA synthesis. *Environ Health Perspect* 40, 197-205 (1981).

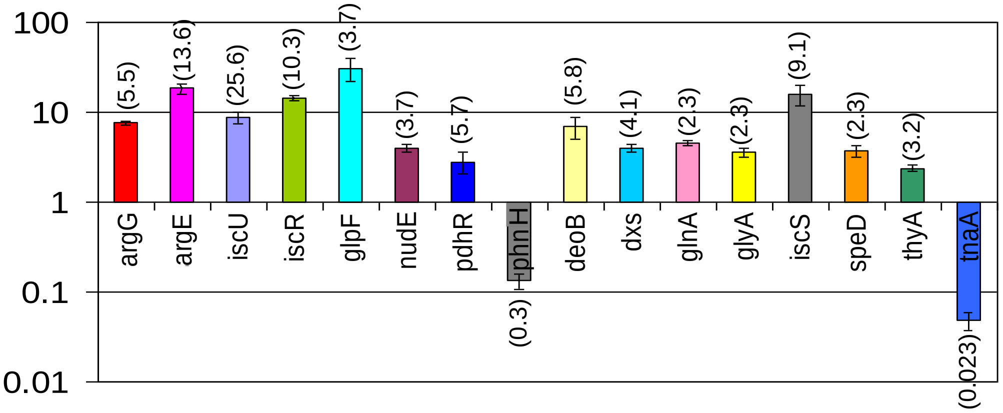

Fig S1. qRT-PCR data validates microarray experiment

Bar diagrams represents manganese-mediated differential expression (fold changes) of the genes, as observed by qRT-PCR experiments. Corresponding differential expression under microarray has been shown in the parenthesis.

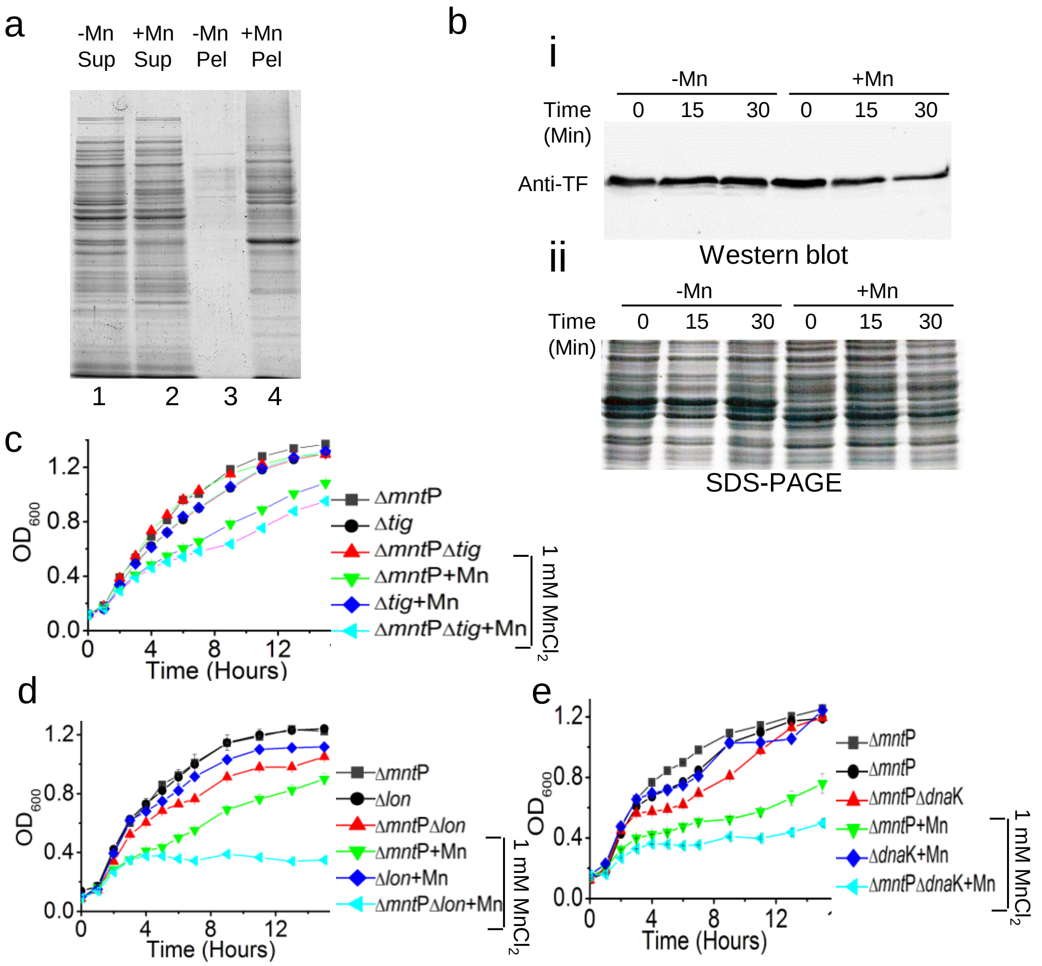

Fig S2. Manganese stress induces protein aggregation and degradation.
(**a**) Coomassie stained SDS-PAGE gel shows that manganese stress promotes protein aggregation or inclusion body formations that appeared in the lane 4; while unstressed cells show a very little inclusion body formation (lane 3). (**b**) Western blotting (ii) shows that managnese exposure to growing Δ*mnt*P cells reduces the level of TF chaperone. The lower panel (ii) is a coomassie-stained gel to show that the equal amounts of protein loaded in each lane for western blotting. (**c**) Growth assays exhibits that Δ*mnt*PΔ*tig* strain is marginally more sensitive than Δ*mnt*P strain under manganese stress. (**d**) and (e) Growth curves exhibit that Δ*mnt*PΔ*lon* and Δ*mnt*PΔ*lon* strains are extremely sensitive than Δ*mnt*P strain under manganese stress.

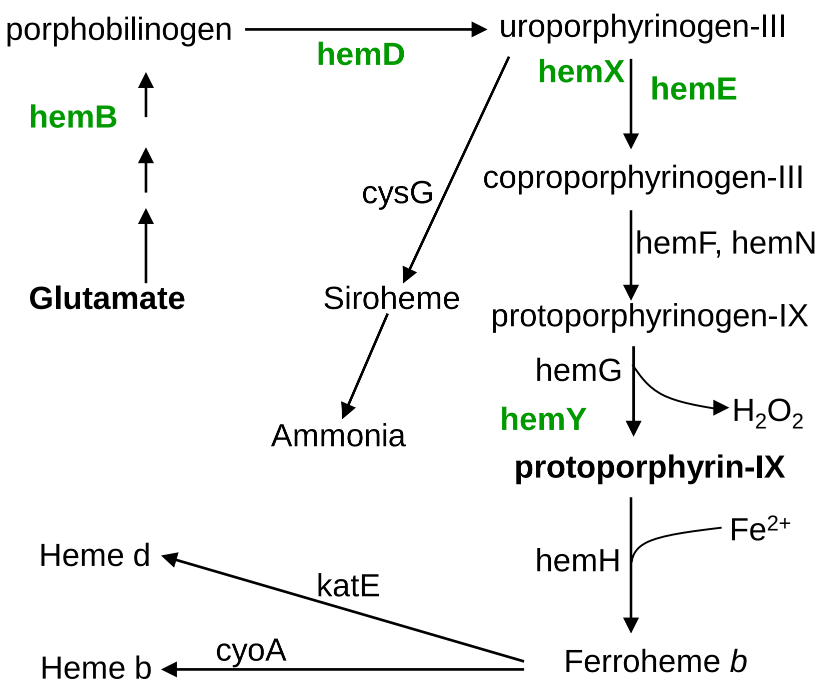

Fig S3. Schematic showing that protoporphyrin synthesis genes are activated

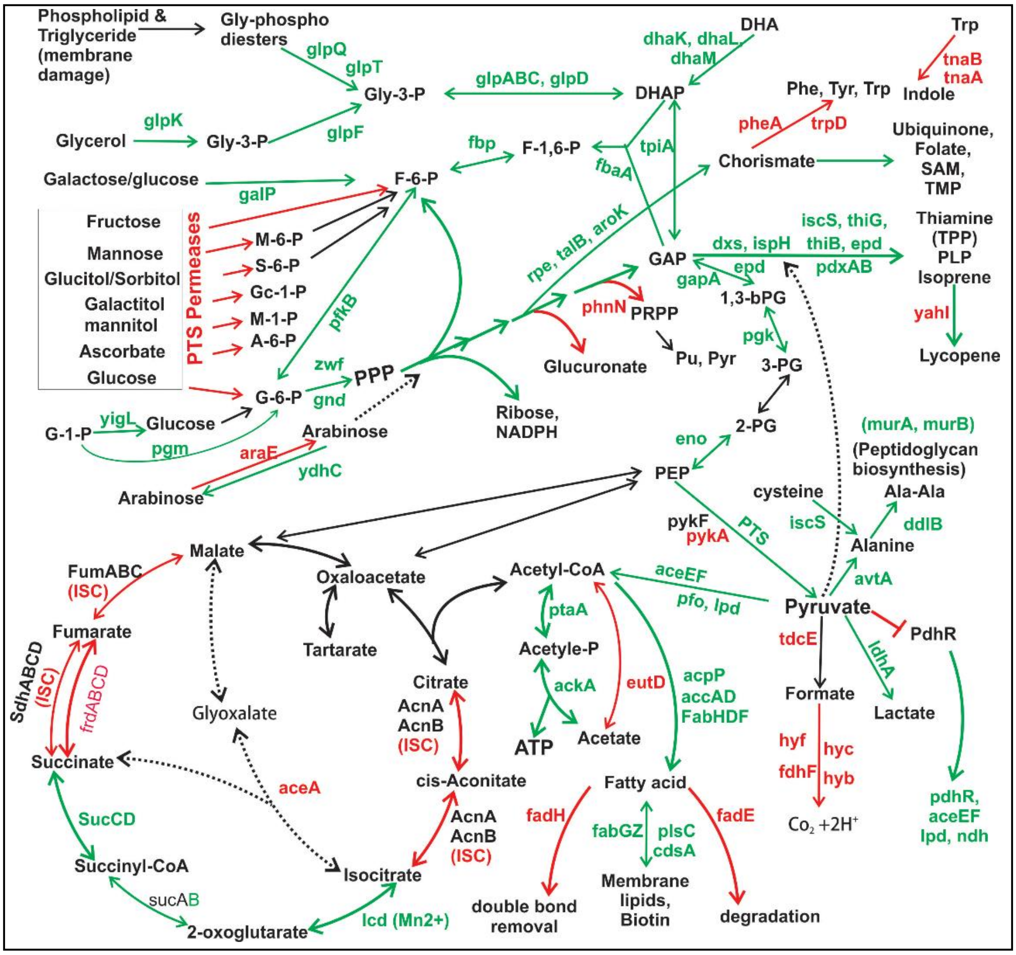

Fig S4. Manganese stress affects carbon metabolism pathways

(A) The pathways represents upregulated (green) and downregulated (red) genes under manganese stress. Fold changes of the designated genes are shown in the Table S4.

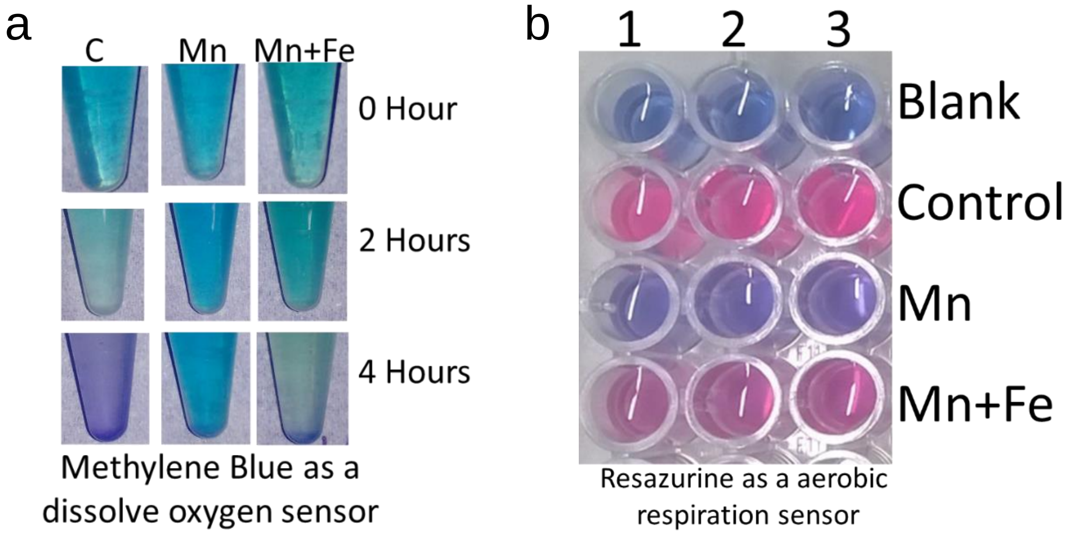

Fig. S5. Indicator dyes exhibit respiratory status under manganese stress.

a. Methylene blue dye turns blue to violate with the time in the control Δ*mntP* cells (manganese-untreated) because dissolved oxygen levels gradually decreases in the sealed microfuge tube with the growth of the cells. On the other hand, color remains unchanged when Δ*mntP* cells were fed with manganese, suggesting that dissolve oxygen consumption reduced. While manganese-fed cells were supplemented with iron, the dissolve oxygen consumption was restored, as indicated by change of the color. **b**. Resazurine dye turns blue to pink in the growing untreated Δ*mntP* or iron-supplemented manganese-fed cells, suggesting that the aerobic respiration does not affected in these conditions. On the contrary, manganese-fed cells does not change the color of the dye, suggesting that manganese stress inhibits aerobic respiration.

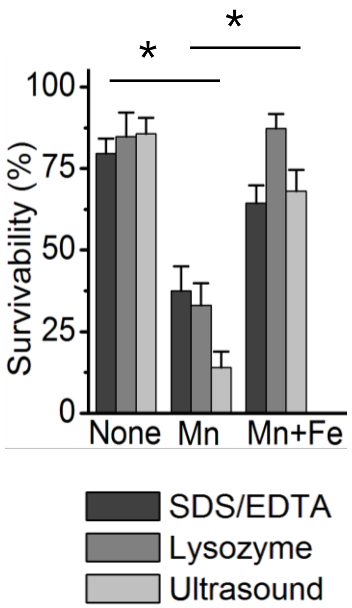

Fig. S6. Manganese stress hinders membrane biogenesis
Survivability of the manganese-fed cells after SDS/EDTA, lysozyme and ultrasound exposure. Iron supplementation increases the survivability. (**D)** The growth curve showing that Δ*mnt*PΔ*icd* strain is more sensitive to manganese than Δ*mnt*P strain. Data (B), (C) and (D) are means ± SD (n=3); *P<0.001, paired T-test.

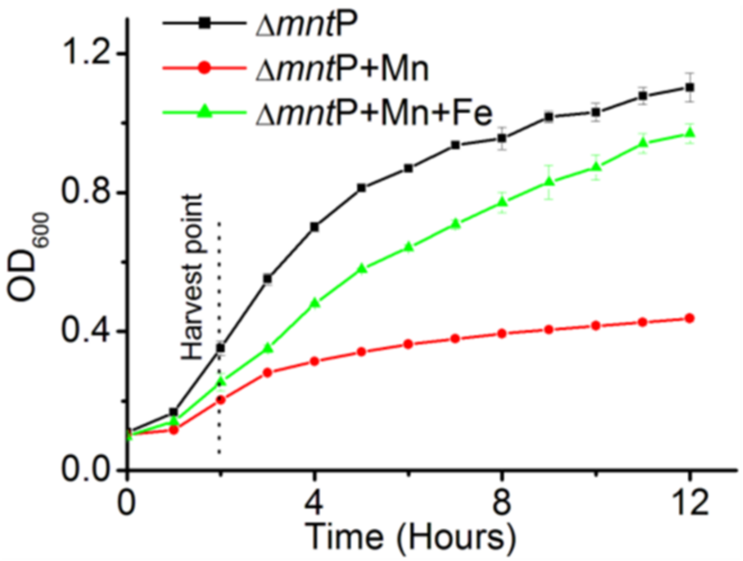

Fig. S7. Growth curve showing the *E. coli* cells harvest point

The dotted line in the growth curve indicates that after 2 hours of secondary growth from a 1:100 dilution of overnight culture in LB, the cells were harvested for mRNA isolation for microarray and real time PCR.

Table S1. Differential expression of the genes for chaperon, proteases and protein transport and cell adhesion and ribosome biogenesis

Gene	Accession ID	Mn F Change	Colour Code	Mn+Fe F Change	Colour Code	
cpxP	b4484	5.380774		1.865571		
dnaJ	b0015	3.5619		1.698284		
dnaK	b0014	3.641517		5.709233		
dsbD	b4136	5.860478		3.156951		
fkpA	b3347	4.070545		2.207245		
groL	b4143	4.263447		2.498905		
groS	b4142	10.62865		2.734571		
grpE	b2614	3.605382		2.600343		
grxC	b3610	5.155962		3.084331		
grxD	b1654	5.17478		1.820911		
hscA	b2526	9.385939		6.938944		
hscB	b2527	15.62294		10.61006		
hslJ	b1379	3.172198		1.748886		
hslO	b3401	4.284679		1.487885		
hslR	b3400	5.187535		2.942911		
hslU	b3931	12.74528		3.69543		Chaperon genes
htpG	b0473	5.339245		3.341361		
hupA	b4000	5.930149		3.366687		
ibpA	b3687	9.118373		1.85146		
ibpB	b3686	3.624389		1.894104		
ldtB	b0819	3.838256		1.527675		
lolA	b0891	3.198597		2.821926		
map	b0168	4.923666		2.977486		
ppiA	b3363	4.337385		2.388791		
ridA	b4243	3.316129		3.742444		
skp	b0178	3.473105		2.394568		
surA	b0053	3.05781		1.449804		
fimC	b4316	0.069482		0.083495		
cbpM	b0999	0.233947		0.496621		
cbpA	b1000	0.274216		0.383045		
hybE	b2992	0.256472		0.402069		
tig	b0436	4.346455		1.970452		
yobF	b1824	4.357549		2.499467		
secA	b0098	3.050489		1.69508		
secB	b3609	3.638787		2.525754		
secG	b3175	3.25405		1.058159		
secY	b3300	5.578128		6.601394		
tatA	b3836	5.72837		4.3683		
yidC	b3705	6.622049		2.969221		
proV	b2677	8.76778		2.734214		
proX	b2679	4.22981		2.455791		
kbl	b3617	3.938732		4.345514		
panF	b3258	3.822482		1.624895		Protein translocation and chemical chaperon maintainance genes
artI	b0863	3.418509		1.42103		
argE	b3957	13.61215		2.591834		
argG	b3172	5.471422		1.656374		
gcvP	b2903	0.184592		0.373498		
gcvH	b2904	0.429049		0.717599		
gcvT	b2905	0.216488		0.291771		
glyA	b2551	2.265547		1.815912		
fimA	b4314	0.051105		0.096573		
fimC	b4316	0.069482		0.083495		
fimF	b4318	0.262758		0.346707		
fimI	b4315	0.095916		0.203205		
sseB	b2522	4.675344		3.935003		
rplB	b3317	5.989547		4.702267		
rplC	b3320	7.295904		4.650516		
rplD	b3319	4.723908		4.205494		Ribosome and its biogenesis genes
rplE	b3308	3.736921		2.661606		
rplF	b3305	7.432805		6.614697		
rplI	b4203	5.533706		4.838303		
rplK	b3983	3.974095		2.235456		
rplM	b3231	11.42815		5.273433		
rplN	b3310	3.684052		2.929786		
rplO	b3301	9.593349		12.17539		

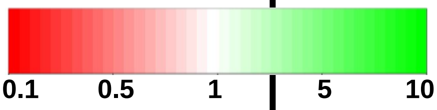

Table S1. (continued)

Gene	Accession ID	Mn F Change	Colour Code	Mn+Fe F Change	Colour Code
rplP	b3313	5.584286		6.069701	
rplQ	b3294	8.117891		4.946949	
rplR	b3304	3.978015		3.360046	
rplT	b1716	6.544902		5.97072	
rplU	b3186	3.748071		1.456896	
rplV	b3315	9.850974		7.654492	
rplW	b3318	4.825051		3.286424	
rplX	b3309	4.788962		3.577738	
rplY	b2185	13.0817		5.612679	
rpmA	b3185	4.184337		1.193586	
rpmB	b3637	10.90018		9.682895	
rpmC	b3312	8.240012		4.914194	
rpmD	b3302	7.391767		8.567037	
rpmE	b3936	8.351967		2.617462	
rpmF	b1089	4.733131		2.181331	
rpmG	b3636	13.24558		13.71036	
rpmJ	b3299	4.103922		3.053994	
rpsA	b0911	7.134047		4.051817	
rpsC	b3314	13.39511		8.385251	
rpsD	b3296	3.76438		5.20726	
rpsE	b3303	5.980102		5.469383	
rpsF	b4200	6.921834		5.737893	
rpsH	b3306	5.73486		4.070454	
rpsI	b3230	8.63232		4.803195	
rpsJ	b3321	8.068261		3.286754	
rpsK	b3297	4.651193		6.355583	
rpsM	b3298	5.440208		8.965576	
rpsN	b3307	5.893087		3.012816	
rpsO	b3165	7.470413		2.790953	
rpsP	b2609	5.285577		3.35454	
rpsQ	b3311	6.558678		4.725864	
rpsR	b4202	6.720849		6.27839	
rpsS	b3316	8.930149		6.446606	
rpsU	b3065	11.88332		6.315573	
rraA	b3929	1.148193		1.197068	
rraB	b4255	4.793195		1.78824	
rrlC	b3758	13.30224		16.0237	
rrlD	b3275	12.10641		15.09942	
rrlE	b4009	9.827944		12.08896	
rrlH	b0204	12.80337		16.22174	
rrsA	b3851	4.433212		7.929275	
rrsC	b3756	4.369206		8.277735	
rrsD	b3278	4.515355		8.180948	
rrsE	b4007	4.508478		8.717388	
rrsG	b2591	4.567053		8.97346	
rrsH	b0201	4.518582		8.116503	
der	b2511	3.191369		1.270542	
dksA	b0145	4.771053		1.786023	
nusG	b3982	7.7766		4.191004	
orn	b4162	3.398171		1.326029	
prmA	b3259	4.015924		1.906995	
rbfA	b3167	4.734938		2.817503	
rimI	b4373	3.600361		1.537542	
rng	b3247	3.423501		1.941022	
rsmG	b3740	9.895999		2.669748	
rsmH	b0082	3.174522		1.649765	
yibL	b3602	3.515828		1.83367	
frr	b0172	5.361664		1.947591	
fusA	b3340	3.112192		1.790934	
infB	b3168	3.25462		2.604027	
prfB	b2891	3.500211		2.333899	
prfC	b4375	9.806155		3.012686	
tsf	b0170	4.123501		3.577185	
tufA	b3339	3.362616		3.328196	
rrlA	b3854	12.43212		15.7327	
rrlB	b3970	11.54735		13.42864	
rimM	b2608	4.404634		6.016764	
rsmB	b3289	4.680956		3.822335	

Ribosome and its biogenesis genes

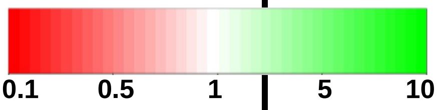

Table S1. (continued)

Gene	Accession ID	Mn F Change	Colour Code	Mn+Fe F Change	Colour Code	
alaS	b2697	3.330342		2.483577		
argS	b1876	6.785676		2.824492		
glyS	b3559	3.155922		2.002968		
glyT	b3978	3.838291		1.747296		
glyU	b2864	2.042816		1.008497		
ileS	b0026	3.760274		2.256425		
leuS	b0642	3.332758		1.825886		
lysS	b2890	4.167147		2.021666		
pheT	b1713	3.011341		3.691313		
trpS	b3384	7.748846		4.169579		tRNA and tRNA synthase genes
valS	b4258	4.676873		2.564365		
asnU	b1986	0.235356		0.450214		
thrV	b3273	3.717036		1.592926		
ileX	b3069	0.337699		0.694154		
leuU	b3174	3.386883		1.454132		
leuZ	b1909	4.862379		1.895104		
trmD	b2607	4.982905		6.894261		
dusB	b3260	3.217662		1.728709		
miaB	b0661	5.020875		1.882271		
trpT	b3761	3.194177		1.733234		
glmS	b3729	4.484252		1.810354		
guaA	b2507	3.565268		1.782194		
hslV	b3932	4.286987		2.159415		
ldtB	b0819	3.838256		1.527675		
lon	b0439	3.883322		2.490988		
map	b0168	4.923666		2.977486		Protease coding genes
pepB	b2523	5.693507		3.774457		
pepQ	b3847	4.00431		4.81397		
prlC	b3498	3.086934		3.430547		
yhbO	b3153	3.68978		3.755114		
hybD	b2993	0.178476		0.369488		
hyaD	b0975	0.063628		0.126326		
pepT	b1127	0.193236		0.306871		
ompT	b0565	0.189941		0.212341		

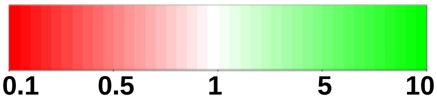

Table S2. Differential expression of the genes in ISC, heme and Moco biosynthesis pathways

Gene	Accession ID	Mn F Change	Colour Code	Mn+Fe F Change	Colour Code	
entA	b0596	0.381446		0.593493		Iron import and Enterobactin Biosynthesis genes
entC	b0593	0.39765		0.656611		
entE	b0594	0.356735		0.633766		
entF	b0586	0.436909		0.634703		
entH	b0597	0.322406		0.631425		
entS	b0591	0.350094		0.607556		
fecB	b4290	0.334288		0.765477		
fecD	b4288	0.378884		0.634218		
fecI	b4293	2.443826		1.642218		
fecR	b4292	0.261207		0.591337		
feoA	b3408	0.437971		1.022697		
fepD	b0590	0.475262		0.521576		
fepG	b0589	0.344871		0.495107		
iscA	b2528	16.32014		16.2128		ISC and heme biogenesis and heme export genes
iscR	b2531	10.35564		6.541137		
iscS	b2530	9.138748		11.97252		
iscU	b2529	25.63876		25.43284		
iscX	b2524	9.133467		5.804918		
yggX	b2962	3.216179		1.68441		
ahpC	b0605	3.187171		2.522515		
hscA	b2526	9.385939		6.938944		
hscB	b2527	15.62294		10.61006		
fdx	b2525	8.711856		5.702443		
nfuA	b3414	2.131591		1.103601		
sufA	b1684	0.604216		1.365996		
sufB	b1683	1.656202		3.043715		
sufC	b1682	1.936803		3.33004		
sufD	b1681	1.06571		3.168184		
sufE	b1679	2.200517		3.464604		
hemB	b0369	3.291861		1.86024		
hemD	b3804	2.601482		2.599917		
hemE	b3997	3.216799		2.503946		
hemX	b3803	3.043231		3.121597		
hemY	b3802	3.063259		2.778651		
ccmA	b2201	0.483357		0.633981		
ccmB	b2200	0.235836		0.414215		
ccmC	b2199	0.4667		0.519369		
ccmD	b2198	0.497914		0.583574		
dps	b0812	0.33366		0.442062		
mnmA	b1133	3.323112		1.501696		tRNA thiolation genes
mnmC	b2324	2.445392		1.477015		
mnmE	b3706	2.019259		2.078851		
mnmG	b3741	13.42164		4.930966		
mnmH	b0503	0.430728		0.49973		
tusD	b3345	2.353549		1.393462		
moaA	b0781	3.897533		2.859393		MoCo Biosynthesis genes
moaB	b0782	11.42286		9.178544		
moaC	b0783	5.746888		4.847944		
moaD	b0784	4.449304		4.244819		
moaE	b0785	5.98877		4.424723		
mocA	b2877	0.288282		0.345944		
modA	b0763	0.487607		0.457255		
modB	b0764	0.426387		0.411045		
mog	b0009	4.715407		1.675826		
thiB	b0068	4.998238		1.680928		Thiamine (Vit-B1) Biosynthesis genes
thiC	b3994	2.830241		1.071211		
thiF	b3992	2.1921		1.095734		
thiG	b3991	3.988686		1.192379		
thiI	b0423	2.653794		0.969189		
thiS	b4407	3.673168		1.341168		
dxs	b0420	4.102929		1.857752		

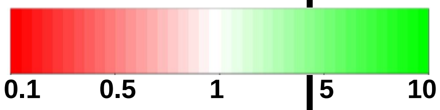

Table S3. Differential expression of the genes of ETC to explain affected energy production and oxidative stress under Mn perturbation

Gene	Accession ID	Mn F Change	Colour Code	Mn+Fe F Change	Colour Code	
ndh	b1109	9.009463		3.554265		NADH dehydrogenase and terminal oxidase associated genes
nuoB	b2287	2.272196		2.386964		
nuoC	b2286	2.370821		3.570267		
nuoE	b2285	2.563837		3.403788		
nuoF	b2284	3.128797		3.147462		
nuoG	b2283	2.675957		2.754937		
nuoK	b2279	2.349189		2.488774		
nuoL	b2278	2.348922		2.044761		
cydC	b0886	4.192281		2.03117		
cydD	b0887	2.47639		1.576809		
cbdA	b0978	0.310291		0.412743		
cbdB	b0979	0.273289		0.357158		
cbdX	b4592	0.175147		0.266642		
glcD	b2979	0.261541		0.638904		
glcE	b4468	0.127225		0.373665		
ubiB	b3835	4.043655		3.505679		Electron carriers ubiquinone and menaquinone synthesizing genes
ubiC	b4039	3.835396		2.361975		
ubiD	b3843	2.603865		5.132762		
ubiE	b3833	3.066901		2.616862		
ubiI	b2906	2.456136		2.128869		
ubiJ	b3834	4.07373		3.341982		
menC	b2261	0.375182		0.698256		
atpA	b3734	8.265748		7.880746		ATP synthase genes
atpB	b3738	3.504014		3.052514		
atpC	b3731	2.471169		2.805655		
atpD	b3732	2.863173		2.558664		
atpE	b3737	1.980756		2.291977		
atpF	b3736	6.30702		3.882043		
atpG	b3733	5.732687		5.900594		
atpH	b3735	9.322741		9.797908		
atpI	b3739	2.021578		1.558663		
napA	b2206	0.281697		0.466478		Nitrogen metabolism and nitrate/nitrite respiration and Xanthine oxidase genes
napD	b2207	0.281083		0.570974		
napF	b2208	0.395582		0.525728		
napH	b2204	0.334408		0.597847		
narG	b1224	0.227507		0.323649		
narH	b1225	0.075795		0.137976		
narJ	b1226	0.23566		0.322051		
narK	b1223	0.099279		0.194464		
narP	b2193	0.414987		0.634621		
narQ	b2469	0.387356		0.625686		
narV	b1465	0.381123		0.662313		
narW	b1466	0.486124		0.820187		
nfsA	b0851	2.791852		1.679085		
nfsB	b0578	2.090862		1.172883		
nirC	b3367	0.36937		0.904267		
nirD	b3366	0.340725		0.855372		
nrfA	b4070	0.097089		0.207087		
nrfB	b4071	0.43794		0.776427		
nrfC	b4072	0.177669		0.453929		
nrfD	b4073	0.125191		0.47906		
cynX	b0341	1.20178		0.186533		
cynS	b0340	2.251557		1.19991		
xdhA	b2866	0.205713		0.354406		
xdhB	b2867	0.12465		0.263682		
xdhC	b2868	0.288649		0.566635		
xanQ	b2882	0.237873		0.46823		
xapB	b2406	0.469446		0.696602		
xapA	b2407	0.46829		0.699316		
paoB	b0285	0.507612		0.849378		
paoC	b0284	0.323376		0.617098		
fdnG	b1474	0.129986		0.307093		

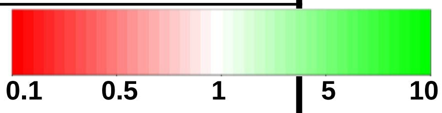

Table S3. (continued)

Gene	Accession ID	Mn F Change	Colour Code	Mn+Fe F Change	Colour Code
dmsA	b0894	0.030929		0.060842	
dmsB	b0895	0.021493		0.040391	
dmsC	b0896	0.16829		0.297633	
dmsD	b1591	0.48035		0.342754	
ynfG	b1589	0.033645		0.05947	
ynfE	b1587	0.013811		0.02384	
ynfF	b1588	0.048681		0.090682	
tehA	b1429	3.931559		2.345492	
tehB	b1430	8.194151		9.018319	
actP	b4067	0.316768		0.82372	
psd	b4160	3.206033		1.791582	
hmp	b2552	11.97481		6.458794	
flgI	b1080	0.062257		0.097256	
flgJ	b1081	0.073959		0.119714	
flgK	b1082	0.060217		0.085066	
flgL	b1083	0.227589		0.257051	
flgM	b1071	0.140443		0.129823	
flgN	b1070	0.201315		0.200972	
flhA	b1879	0.247124		0.387706	
flhB	b1880	0.326549		0.592462	
flhC	b1891	0.208805		0.287053	
flhD	b1892	0.284627		0.303667	
flhE	b1878	0.415074		0.479738	
fliA	b1922	0.074758		0.083668	
fliC	b1923	0.095361		0.015228	
fliD	b1924	0.044771		0.039613	
fliE	b1937	0.284165		0.534829	
fliF	b1938	0.314105		0.492274	
fliG	b1939	0.033709		0.050285	
fliH	b1940	0.077432		0.146553	
fliI	b1941	0.045898		0.074525	
fliJ	b1942	0.068416		0.127055	
fliK	b1943	0.038725		0.060719	
fliL	b1944	0.140716		0.169197	
fliM	b1945	0.046524		0.051358	
fliN	b1946	0.041518		0.048974	
fliO	b1947	0.278614		0.32214	
fliP	b1948	0.274183		0.374231	
fliQ	b1949	0.098482		0.201679	
fliR	b1950	0.23923		0.390037	
fliS	b1925	0.038707		0.031796	
fliT	b1926	0.032788		0.01658	
fliZ	b1921	0.043623		0.053405	
motA	b1890	0.10038		0.112536	
motB	b1889	0.070016		0.014858	
ycgR	b1194	0.392501		0.398975	
cheA	b1888	0.098377		0.072783	
cheB	b1883	0.25043		0.380042	
cheR	b1884	0.13444		0.175108	
cheW	b1887	0.108646		0.043056	
cheY	b1882	0.392631		0.328634	
cheZ	b1881	0.10568		0.132105	
tsr	b4355	0.224013		0.325621	
aer	b3072	0.259314		0.481388	
tap	b1885	0.082167		0.094531	
tar	b1886	0.089902		0.122011	
bdm	b1481	3.041796		2.176663	
yhjH	b3525	0.22962		0.533384	
flk	b2321	3.533169		1.511317	

DMSO and selenate reductase, and tellurite efflux genes

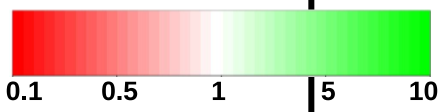

Flagella biogenesis and motility-associated genes

Table S3. (continued)

Gene	Accession ID	Mn F Change	Colour Code	Mn+Fe F Change	Colour Code
ychF	b1203	3.565408		1.480669	
ndh	b1109	9.009463		3.554265	
glnA	b3870	2.255503		1.266069	
sodA	b3908	16.19521		14.53883	
sodC	b1646	0.229828		0.444379	
soxS	b4062	3.762688		1.656495	
fur	b0683	3.2775		1.653435	
nfsA	b0851	2.791852		1.679085	
nfsB	b0578	2.090862		1.172883	
fpr	b3924	4.765849		4.554986	
rsxB	b1628	3.720381		1.084946	
rsxC	b1629	5.035981		1.607547	
rsxD	b1630	2.619001		1.177383	
rsxE	b1632	2.466241		1.061105	
rsxG	b1631	2.015258		1.084759	
ahpC	b0605	3.187171		2.522515	
ahpF	b0606	4.489267		1.773706	
ridA	b4243	3.316129		3.742444	
gor	b3500	3.466474		2.886765	
grxC	b3610	5.155962		3.084331	
grxD	b1654	5.17478		1.820911	
trxB	b0888	4.278193		2.344977	
alx	b3088	3.195605		0.951694	
bisC	b3551	2.042498		2.112128	
cydC	b0886	4.192281		2.03117	
cydD	b0887	2.47639		1.576809	
gshA	b2688	2.196481		1.32534	
cysH	b2762	2.202875		1.873322	
cysJ	b2764	2.511702		2.389997	
cysK	b2414	4.112354		2.568663	
cysS	b0526	2.008311		1.148733	
cysT	b1910	6.068874		2.209115	
dfp	b3639	6.144095		3.141423	
coaA	b3974	2.289919		1.422695	
tnaA	b3708	0.022674		0.137114	
tpx	b1324	2.713464		1.6222	

Oxidative stress, redox Response and cysteine metabolism genes genes

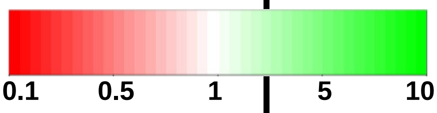

Table S4. Differential expression of the genes in carbon metabolism networks

Glycerol uptake and metabolism genes

Gene	Accession ID	Mn F Change	Colour Code	Mn+Fe F Change	Colour Code
glpA	b2241	3.692059		2.469699	
glpB	b2242	3.495898		3.380901	
glpC	b2243	3.810216		3.602358	
glpD	b3426	29.44443		10.64017	
glpF	b3927	3.730397		7.112676	
glpK	b3926	4.393517		8.715906	
glpQ	b2239	3.849661		5.589229	
glpT	b2240	2.65854		3.088363	
dhaK	b1200	2.70314		0.848994	
dhaL	b1199	2.690491		1.154747	
dhaM	b1198	5.642882		0.78484	

Sugar transport, gluoconeogenesis and pentose phosphate pathway genes

Gene	Accession ID	Mn F Change	Colour Code	Mn+Fe F Change	Colour Code
manA	b1613	0.376145		0.333162	
manX	b1817	0.231454		0.26874	
manY	b1818	0.214984		0.156643	
manZ	b1819	0.350504		0.505067	
gatA	b2094	0.182142		0.452336	
gatB	b2093	0.168268		0.440489	
gatC	b2092	0.157928		0.359008	
gatD	b2091	0.12903		0.279053	
gatR	b4498	0.276036		0.621779	
gatY	b2096	0.152591		0.285122	
gatZ	b2095	0.179444		0.397857	
srlA	b2702	0.08921		0.187021	
srlB	b2704	0.100797		0.258201	
srlD	b2705	0.068337		0.12433	
srlE	b2703	0.130908		0.264257	
srlM	b2706	0.084528		0.150993	
srlQ	b2708	0.315798		0.597086	
srlR	b2707	0.357346		0.454391	
frvB	b3899	0.146761		0.595379	
frwC	b3949	0.342087		0.944224	
frwD	b3953	0.324645		0.98903	
agaA	b3135	0.210037		0.56664	
agaC	b3139	0.397599		0.659448	
agaS	b3136	0.385032		0.714313	
agaW	b3134	0.24038		0.671964	
cmtA	b2933	0.264938		0.601219	
sgcA	b4302	0.375464		0.785805	
sgcB	b4565	0.175877		0.386772	
sgcR	b4300	0.307263		0.797716	
sgcX	b4305	0.411223		0.73223	
ulaA	b4193	0.345945		0.867216	
ulaE	b4197	0.499987		1.070613	
mdtD	b2077	0.322404		0.562035	
ydhC	b1660	6.296473		0.593405	
crr	b2417	2.72856		2.611643	
ptsG	b1101	0.560091		0.575806	
sgrS	b4577	3.942012		1.210146	
sgrT	b4662	4.157273		1.138825	
aroK	b3390	2.516364		0.784845	
tnaA	b3708	0.022674		0.137114	
tnaB	b3709	0.016847		0.07337	
tnaC	b3707	0.059066		0.094119	
mtr	b3161	0.331695		0.678999	
eno	b2779	2.924622		2.49205	
pgk	b2926	2.244046		1.896238	
tpiA	b3919	2.311995		3.535583	
fbaA	b2925	4.281778		3.973715	
fbp	b4232	3.715784		2.689692	
gapA	b1779	2.2821		3.611816	
pfkB	b1723	3.26588		2.86773	
pgm	b0688	2.001915		1.313448	
yigL	b3826	2.937694		1.727054	
talB	b0008	4.412729		3.10397	
zwf	b1852	4.553325		3.093566	
gnd	b2029	3.343018		2.039286	

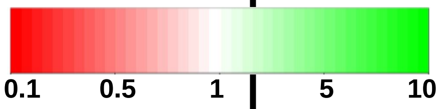

Table S4. (continued)

Gene	Accession ID	Mn F Change	Colour Code	Mn+Fe F Change	Colour Code	
rpe	b3386	3.824665		2.408429		Sugar transport, gluoconeogenesis and pentose phosphate pathway genes
ghrB	b3553	10.29303		9.75737		
pykA	b1854	0.325511		0.429123		
phnN	b4094	0.344567		0.946868		
uxaB	b1521	0.273283		0.433843		
uxaA	b3091	0.171493		0.362107		
uxaC	b3092	0.252623		0.658991		
uxuB	b4323	0.148066		0.295855		
ytfT	b4230	0.300192		0.645319		
garP	b3127	0.340664		0.80099		
paaG	b1394	0.228765		0.487038		
rbsB	b3751	0.271926		0.553185		
galP	b2943	3.473264		1.737665		
ugpA	b3452	0.251374		0.711925		
ugpE	b3451	0.337165		0.913487		
exuT	b3093	0.108211		0.216882		
mhpA	b0347	0.339156		0.778296		
cdaR	b0162	0.274611		0.53578		
xylH	b3568	0.352		0.898336		
mhpB	b0348	0.150394		0.45041		
mhpT	b0353	0.116454		0.385853		
prpE	b0335	0.140447		0.407101		
gntP	b4321	0.349609		0.683162		
gntU	b4476	0.315833		0.984103		
eutG	b2453	0.152469		0.409212		
eutM	b2457	0.260902		0.666287		
eutQ	b2460	0.173651		0.469305		
mak	b0394	0.319634		0.616632		
ttdR	b3060	0.041664		0.101587		
mtr	b3161	0.331695		0.678999		
thiB	b0068	4.998238		1.680928		Thiamine and Isoprenoid, PLP biosynthesis genes
thiC	b3994	2.830241		1.071211		
thiF	b3992	2.1921		1.095734		
thiG	b3991	3.988686		1.192379		
thiI	b0423	2.653794		0.969189		
thiS	b4407	3.673168		1.341168		
dxs	b0420	4.102929		1.857752		
epd	b2927	3.36622		1.915362		
pdxA	b0052	2.136396		1.155329		
pdxB	b2320	2.492974		2.011509		
ispB	b3187	2.457049		1.236322		
ispG	b2515	2.32115		1.624895		
ispH	b0029	3.211278		2.487319		
yahI	b0323	0.260894		0.658967		
ndh	b1109	9.009463		3.554265		Phosphotransferase system and Pyruvate metabolism and TCA cycle genes
pdhR	b0113	5.695002		1.836674		
aceE	b0114	8.058289		10.43817		
aceF	b0115	10.55611		10.0587		
ptsH	b2415	2.358706		1.903847		
ptsN	b3204	2.532791		2.592674		
ptsP	b2829	2.149981		1.338608		
lpd	b0116	8.062529		8.504626		
accA	b0185	4.277306		2.097398		
pfo	b1378	6.549534		3.218996		
ldhA	b1380	9.29784		4.919991		
ackA	b2296	3.425421		2.245204		
accD	b2316	3.865538		1.961356		
pta	b2297	2.91002		1.916223		
sucB	b0727	2.74781		2.752843		

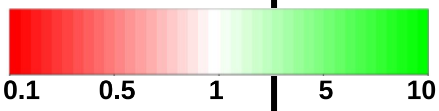

Table S4. (continued)

Gene	Accession ID	Mn F Change	Colour Code	Mn+Fe F Change	Colour Code	
eutD	b2458	0.259764		0.60001		Hydrogenase, **nickel transport** and TCA cycle associated genes
hybA	b2996	0.03445		0.039794		
hybB	b2995	0.152738		0.28709		
hybC	b2994	0.190007		0.358362		
hybD	b2993	0.178476		0.369488		
hybE	b2992	0.256472		0.402069		
hybF	b2991	0.132752		0.228778		
hybG	b2990	0.171094		0.19744		
hybO	b2997	0.044776		0.068472		
hycB	b2724	0.30885		0.733712		
hycC	b2723	0.225234		0.551107		
hycD	b2722	0.251047		0.54437		
hycE	b2721	0.281558		0.589291		
hyfE	b2485	0.309901		0.55969		
hyaA	b0972	0.126143		0.199797		
hyaB	b0973	0.318498		0.440318		
hyaC	b0974	0.104944		0.255273		
hyaE	b0976	0.175089		0.324025		
hyaF	b0977	0.158388		0.280263		
hypA	b2726	0.298813		0.663686		
hypB	b2727	0.098906		0.247444		
hypC	b2728	0.097823		0.21751		
hypD	b2729	0.080708		0.18281		
hypE	b2730	0.085597		0.15433		
hypF	b2712	0.328072		0.621204		
fdhF	b4079	0.217773		0.552479		TCA cycle and pyruvate and dicarboxylate transport associated genes
tdcA	b3118	0.283361		0.432897		
tdcC	b3116	0.023637		0.068635		
tdcD	b3115	0.015708		0.028758		
tdcF	b3113	0.025542		0.111882		
tdcG	b4471	0.066681		0.256659		
frdA	b4154	0.145953		0.382506		
frdB	b4153	0.121587		0.247255		
frdC	b4152	0.133882		0.314911		
frdD	b4151	0.100362		0.170924		
fumB	b4122	0.049689		0.109277		
dcuB	b4123	0.072591		0.182187		
dcuC	b0621	0.075907		0.130704		
dcuR	b4124	3.169232		1.859637		

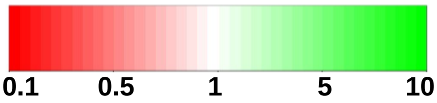

Table S5. Differential expression profile of the genes in amino acid metabolism

Gene	Accession ID	Mn F Change	Colour Code	Mn+Fe F Change	Colour Code	
argC	b3958	2.500933		1.40263		Amino acid metabolism genes
argE	b3957	13.61215		2.591834		
argG	b3172	5.471422		1.656374		
argH	b3960	1.934712		1.132867		
argO	b2923	0.368134		0.620176		
argR	b3237	3.236021		1.734606		
carA	b0032	3.993839		1.117164		
carB	b0033	2.956424		0.911363		
artI	b0863	3.418509		1.42103		
artJ	b0860	2.600965		0.973015		
artQ	b0862	2.535432		1.077086		
lysP	b2156	4.047354		0.567834		
adiC	b4115	0.111369		0.098812		
yahN	b0328	0.210528		0.47121		
cadA	b4131	0.217546		0.163066		
cadB	b4132	0.26329		0.117251		
speA	b2938	2.257532		1.07719		
speC	b2965	2.766847		1.175613		
speD	b0120	2.250701		1.141767		
speE	b0121	3.492352		1.661472		
speG	b1584	0.288473		0.218969		
hemB	b0369	3.291861		1.86024		
hemD	b3804	2.601482		2.599917		
hemE	b3997	3.216799		2.503946		
hemX	b3803	3.043231		3.121597		
hemY	b3802	3.063259		2.778651		
glnA	b3870	2.255503		1.266069		
glmS	b3729	4.484252		1.810354		
glmU	b3730	5.65388		2.712275		
murA	b3189	5.155026		2.115578		
murB	b3972	3.278129		1.750834		
murE	b0085	2.50037		1.62588		
murI	b3967	2.350631		2.531677		
murQ	b2428	0.485447		0.749258		
murR	b2427	0.323054		0.529869		
gltB	b3212	0.355332		0.379281		
glsA	b0485	0.351565		0.66817		
gadA	b3517	0.19292		0.610173		
gadB	b1493	0.218671		0.664332		
gadC	b1492	0.240876		0.551845		
gabD	b2661	0.229184		0.702331		
gabP	b2663	0.366913		0.917989		
puuC	b1300	0.282245		0.526151		
potD	b1123	3.251761		1.677202		
potI	b0857	0.332655		0.528817		
def	b3287	4.861378		2.565936		
pyrB	b4245	3.967528		2.967201		
pyrC	b1062	2.809979		1.506171		
pyrD	b0945	2.17413		0.87558		
pyrE	b3642	0.344466		1.031656		
pyrG	b2780	3.398641		1.831435		
pyrH	b0171	2.222569		1.447102		
pyrI	b4244	12.74772		6.894533		
pyrL	b4246	3.595333		1.202015		
panD	b0131	2.26968		0.775449		
ansB	b2957	0.050486		0.079398		
folA	b0048	2.741889		1.594262		Folate biosynthesis And SAM cycle Genes to produce S-adenosyl Methionine
folE	b2153	3.576133		2.325954		
folX	b2303	4.45081		2.382034		
nudB	b1865	4.666287		2.227312		
gcvP	b2903	0.184592		0.373498		
gcvH	b2904	0.429049		0.717599		
gcvT	b2905	0.216488		0.291771		
lpd	b0116	8.062529		8.504626		
glyA	b2551	2.265547		1.815912		
luxS	b2687	3.059465		1.75311		
mtn	b0159	2.570391		2.13588		
map	b0168	4.923666		2.977486		
metK	b2942	3.414805		2.725421		
speD	b0120	2.250701		1.141767		
ubiE	b3833	3.066901		2.616862		
queF	b2794	2.010878		1.207783		
queG	b4166	2.453106		1.751347		
tgt	b0406	4.752168		1.613502		
thyA	b2827	3.223966		1.3012		

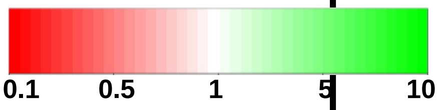

Table S6. Differential expression of the genes in the DNA replication and repair, cell division, transcription pathways

Gene	Accession ID	Mn F Change	Colour Code	Mn+Fe F Change	Colour Code	
dnaB	b4052	2.998618		2.356807		Replication associate Genes
dnaC	b4361	3.651853		1.737679		
dnaE	b0184	3.661848		1.890332		
gyrA	b2231	4.655638		2.149585		
gyrB	b3699	4.69439		3.165146		
holD	b4372	5.093962		1.915185		
hupA	b4000	5.930149		3.366687		
dinD	b3645	5.359852		3.308459		
dut	b3640	8.460442		3.559813		
gyrA	b2231	4.655638		2.149585		
gyrB	b3699	4.69439		3.165146		
hupA	b4000	5.930149		3.366687		
mutL	b4170	4.330019		4.515677		
nfi	b3998	4.006766		3.405689		
nrdA	b2234	20.77903		23.93918		
nrdB	b2235	32.44274		20.18744		
nrdE	b2675	8.492255		10.30535		
nrdF	b2676	3.458012		2.816936		
nrdH	b2673	5.03582		3.620225		
nrdI	b2674	3.632439		4.949042		
nth	b1633	3.041036		1.184243		
polB	b0060	5.150339		1.858043		
priB	b4201	6.645979		6.209072		
recA	b2699	16.30414		8.022686		
recF	b3700	4.001792		2.305637		
recN	b2616	6.286974		2.648384		
recX	b2698	12.80518		4.301149		
ruvA	b1861	4.200069		2.31249		
umuC	b1184	4.171062		2.312513		
uvrA	b4058	6.395952		2.914315		
uvrB	b0779	3.590826		2.473294		
uvrD	b3813	3.287628		2.592532		
yebG	b1848	3.332377		1.302231		
tatD	b4483	3.159446		2.337726		
intE	b1140	3.080642		3.420471		
rmuC	b3832	4.166479		6.343253		
gph	b3385	3.116297		1.934356		
ftsK	b0890	3.023185		1.684591		Cell division associated Genes
ftsL	b0083	3.071722		1.625154		
hupA	b4000	5.930149		3.366687		
mnmG	b3741	13.42164		4.930966		
mreB	b3251	3.520896		1.597333		
mukE	b0923	3.004558		1.566317		
rsmG	b3740	9.895999		2.669748		
slmA	b3641	4.832542		2.219235		
sulA	b0958	7.767213		6.953791		
yihA	b3865	6.192793		3.244912		
zapA	b2910	3.385688		1.584222		
zapB	b3928	8.19753		3.563726		
zapC	b0946	0.131306		0.176931		
damX	b3388	3.725764		2.390354		
deoA	b4382	3.082845		1.162726		Nucleotide catabolism
deoB	b4383	5.790148		4.823739		
deoC	b4381	3.207184		1.506891		
deoD	b4384	5.607811		3.155377		
cdd	b2143	2.235504		0.849213		
adhE	b1241	0.392813		0.402571		
rutA	b1012	0.403118		0.428812		
rutG	b1006	0.374109		0.601806		
tsx	b0411	3.012765		0.988987		Purine and pyrimidine Transport and salvage
nupG	b2964	3.356912		1.016495		
yrfG	b3399	3.330048		1.576189		
pnp	b3164	3.290135		2.203156		
ygfQ	b4464	0.221424		0.527367		
hpt	b0125	3.496292		1.494459		
yfaE	b2236	34.95008		26.00543		
preA	b2147	0.081478		0.214116		
apt	b0469	3.311231		1.052265		
gmk	b3648	3.460641		2.821416		

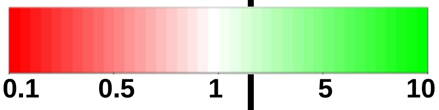

Table S6. (continued)

Gene	Accession ID	Mn F Change	Colour Code	Mn+Fe F Change	Colour Code	
dksA	b0145	4.771053		1.786023		RNAP and associated genes
greA	b3181	4.041358		1.059419		
nusG	b3982	7.7766		4.191004		
rapA	b0059	3.012144		1.528007		
rho	b3783	3.729978		2.24162		
rpoA	b3295	6.418662		5.269561		
rpoB	b3987	9.038267		5.327336		
rpoC	b3988	3.629869		3.841104		
rpoD	b3067	11.08398		3.880112		
rpoN	b3202	3.02744		2.474825		
argR	b3237	3.236021		1.734606		
asnC	b3743	3.453562		1.703798		
crp	b3357	3.162449		2.950174		
cspC	b1823	5.263089		2.660692		
dksA	b0145	4.771053		1.786023		
fur	b0683	3.2775		1.653435		
iscR	b2531	10.35564		6.541137		
marA	b1531	6.198477		4.887065		
pdhR	b0113	5.695002		1.836674		
slmA	b3641	4.832542		2.219235		
soxS	b4062	3.762688		1.656495		
fliA	b1922	0.074758		0.083668		
flhC	b1891	0.208805		0.287053		
flhD	b1892	0.284627		0.303667		
uhpA	b3669	0.191447		0.567308		Two component genes
uhpB	b3668	0.211239		0.747031		
cheA	b1888	0.098377		0.072783		
cheB	b1883	0.25043		0.380042		
phoP	b1130	0.127493		0.163187		
evgA	b2369	0.153889		0.107333		
atoC	b2220	0.170386		0.376684		

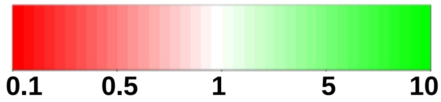

Table S7. Differential expression of the genes for envelop biogenesis

Gene	Accession ID	Mn F Change	Colour Code	Mn+Fe F Change	Colour Code
**Lipids, lipopolysachharide and other cell wall component genes**					
hldD	b3619	4.369055		1.914519	
waaB	b3628	8.840264		2.669181	
waaG	b3631	4.262307		3.718984	
waaJ	b3626	7.449353		3.122623	
waaL	b3622	3.096449		2.329438	
waaP	b3630	7.382194		2.18456	
waaQ	b3632	3.052478		1.589913	
waaR	b3627	8.600487		3.249325	
waaS	b3629	6.164087		2.422539	
waaU	b3623	5.990269		2.811524	
waaY	b3625	4.731342		3.036701	
waaZ	b3624	5.683598		1.707507	
wbbJ	b2033	4.299163		1.671966	
wecB	b3786	3.291863		2.325024	
wzxB	b2037	4.424928		2.19328	
accA	b0185	4.277306		2.097398	
accD	b2316	3.865538		1.961356	
fabD	b1092	2.12554		1.585214	
fabF	b1095	2.135894		1.458666	
fabH	b1091	2.974462		2.117367	
fabG	b1093	4.561977		4.167618	
fabZ	b0180	6.010145		3.223298	
plsC	b3018	4.434031		2.050589	
avtA	b3572	2.381323		1.926011	
ddlB	b0092	3.071785		1.836044	
mreB	b3251	3.520896		1.597333	
mreC	b3250	2.707536		1.871791	
murA	b3189	5.155026		2.115578	
murB	b3972	3.278129		1.750834	
murE	b0085	2.50037		1.62588	
murI	b3967	2.350631		2.531677	
murQ	b2428	0.485447		0.749258	
murR	b2427	0.323054		0.529869	
osmB	b1283	2.397642		1.50745	
osmY	b4376	3.643029		6.715907	
ompT	b0565	0.188841		0.212341	
ompW	b1256	0.18473		0.328108	
ompX	b0814	3.459729		1.555757	
lpp	b1677	3.733236		2.141934	
mlc	b1594	0.195019		0.275297	
mltC	b2963	4.217899		2.060317	
mioC	b3742	11.86136		4.681614	
nlpI	b3163	4.681417		2.439583	
bcsZ	b3531	0.294149		0.913488	
wecG	b3794	3.012059		2.251194	
gmd	b2053	4.626167		1.421919	
fadH	b3081	0.303675		0.85167	
accA	b0185	4.277306		2.097398	
cdsA	b0175	3.005775		1.3931	
wecF	b4481	3.841194		2.861131	
lipA	b0628	5.101534		2.351787	
bamE	b2617	3.341158		1.626087	
rfbC	b2038	5.528325		2.270195	
rfbD	b2040	3.861082		1.506565	
tisB	b4618	8.923626		5.088875	
yidD	b4557	6.49047		2.275864	
dcrB	b3472	6.945913		4.055484	
arnE	b4544	0.327656		0.634324	
**Phosphate recycling related genes**					
yfcD	b2299	4.67835		2.335381	
yjjG	b4374	4.398533		1.874309	
yggF	b2930	0.106674		0.276307	
appA	b0980	0.15377		0.245228	
pstB	b3725	3.435694		2.213305	
gpp	b3779	3.302347		2.186335	
ppa	b4226	4.52296		1.980806	

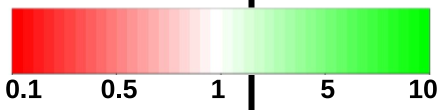

Table S8. Other genes that are differentially expressed

Gene	Accession ID	Mn F Change	Color Code	Mn+Fe F Change	Color Code
ydhT	b1669	0.205874		0.358102	
ydhW	b1672	0.155666		0.318359	
ydhX	b1671	0.125983		0.227732	
ydhY	b1674	0.015357		0.024076	
ynfH	b1590	0.112814		0.270986	
ygfK	b2878	0.153189		0.397925	
wrbA	b1004	0.120721		0.361593	
ycjQ	b1313	0.332612		0.457593	
ydbC	b1406	0.257885		0.494483	
ydgJ	b1624	3.565023		1.786814	
ydhV	b1673	0.064342		0.116721	
ygcN	b2766	0.244964		0.242002	
yghA	b3003	3.687081		3.576489	
yjjK	b4391	3.362874		2.897297	
ynjC	b1755	0.239702		0.493084	
bssS	b1060	0.325949		0.216496	
gadX	b3516	3.234477		2.033998	
uacT	b2888	0.193711		0.447025	
dedA	b2317	3.500347		4.120249	
livH	b3457	0.154935		0.470151	
mgtA	b4242	0.330535		0.675509	
zntA	b3469	0.274338		0.729	
mgrB	b1826	0.324202		0.231405	
afuB	b0263	0.340586		0.539993	
rnpA	b3704	5.64617		1.853726	
marB	b1532	4.635445		4.464833	
yedF	b1930	0.177271		0.308244	
ydaN	b1342	0.220049		0.35696	
caiE	b0035	0.285846		0.632377	
caiF	b0034	0.271285		0.305739	
ddpB	b1486	0.332619		0.640126	
dgoA	b4477	0.184715		0.872855	
dgoD	b4478	0.169694		0.348696	
dkgA	b3012	3.42287		3.352732	
dppC	b3542	0.277054		1.078761	
dtpB	b3496	0.257609		0.397076	
ebgA	b3076	0.255737		0.696061	
essQ	b1556	0.308948		0.504063	
flxA	b1566	0.017107		0.013406	
frlC	b4474	0.305918		0.896885	
frlD	b3374	0.273624		0.742469	
frmB	b0355	3.961009		2.395966	
garL	b3126	0.218615		0.774882	
gtrB	b2351	3.850118		1.28196	
hflC	b4175	3.042627		3.299371	
hflK	b4174	3.45699		3.184261	
hokB	b4428	0.268821		0.459327	
ibaG	b3190	3.638767		1.530492	
insC1	b3044	0.163959		0.528759	
insD1	b1402	0.148079		0.447838	
insD1	b1578	0.148079		0.447838	
insF1	b2089	0.117073		0.289998	
insI1	b0256	0.175123		0.277487	
insI1	b4284	0.175123		0.277487	
insI1	b4708	0.175123		0.277487	
ldrA	b4419	0.225924		0.456317	
ldrB	b4421	0.173307		0.409194	
ldrD	b4453	0.316748		0.852456	
lhgO	b2660	0.349797		1.106851	
lsrA	b1513	0.283264		0.590064	
lsrD	b1515	0.343819		0.682228	
mdtD	b2077	0.322404		0.562035	
mdtM	b4337	0.256305		0.657055	
mdtN	b4082	0.177642		0.662088	
melA	b4119	0.243374		0.771385	
melB	b4120	0.248548		0.465708	
mltD	b0211	4.623187		1.138503	
mntS	b4705	0.077831		0.112578	
mokB	b1420	0.305626		0.454461	
mtfA	b1976	0.245824		0.482056	

Some other ferrodoxin and oxidoreductases

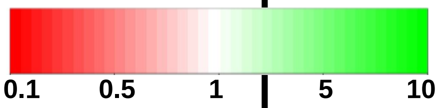

Table S8. (continued)

Gene	Accession ID	Mn F Change	Color Code	Mn+Fe F Change	Color Code
nepI	b3662	0.262763		0.938725	
ogrK	b2082	3.329894		1.799391	
oppA	b1243	0.31857		0.30601	
phnE	b4104	0.137877		0.536988	
phnG	b4101	0.344394		0.894068	
phnH	b4100	0.233918		0.558188	
phnI	b4099	0.21745		0.713422	
phnK	b4097	0.312093		0.886892	
pntB	b1602	0.252901		0.272841	
prfH	b0236	0.340412		0.682012	
rbbA	b3486	0.263256		0.783151	
ryjB	b4624	0.244852		0.548466	
ssrA	b2621	4.790214		5.383299	
ssrS	b2911	3.563847		5.010803	
tauC	b0367	0.323543		0.638672	
tff	b4414	4.976331		1.628582	
uhpC	b3667	0.27169		0.685084	
uhpT	b3666	0.155927		0.430467	
usg	b2319	3.085398		1.777045	
uspC	b1895	0.245076		0.3922	
uspF	b1376	0.292529		0.363536	
viaA	b3745	0.229517		0.418663	
yafN	b0232	4.221624		2.155027	
yafO	b0233	3.13273		1.847243	
yafZ	b0252	0.213783		0.385318	
yagH	b0271	0.137388		0.359713	
yagU	b0287	0.326685		0.270005	
yahK	b0325	0.327299		0.409766	
yahO	b0329	0.309792		0.332517	
yaiI	b0387	0.309717		0.538233	
ybaT	b0486	0.21222		0.420835	
ybaV	b0442	0.280473		0.263618	
ybbP	b0496	0.349142		0.380649	
ybeD	b0631	3.007428		1.05781	
ybeL	b0643	0.13411		0.218561	
ybfD	b0706	0.319792		0.569128	
ybgJ	b0711	0.310822		0.593821	
ybhG	b0795	0.318175		0.521389	
ybhI	b0770	0.247576		0.464399	
ybhQ	b0791	0.349298		0.643667	
ybiI	b0803	0.136793		0.328601	
ybiJ	b0802	6.693067		0.976196	
ybiY	b0824	0.153025		0.250442	
yccJ	b1003	0.317831		0.639384	
ycgG	b1168	0.335689		0.259403	
ycgI	b4521	0.3154		0.557817	
yciN	b1273	3.198423		1.31964	
ydcJ	b1423	0.184303		0.506407	
ydcS	b1440	0.350077		0.719337	
ydcT	b1441	0.269073		0.710143	
ydcV	b1443	0.15542		0.433895	
ydeN	b1498	0.304203		0.642727	
ydhJ	b1644	0.342888		0.360967	
ydiH	b1685	0.16604		0.181681	
ydiP	b1696	0.283647		0.389489	
ydiU	b1706	4.494675		3.574498	
ydjY	b1751	0.282239		0.49123	
ydjZ	b1752	0.196983		0.310393	
yeaG	b1783	0.337458		1.132168	
yebC	b1864	3.154382		1.483274	
yebF	b1847	4.3383		2.367286	
yebQ	b1828	0.282235		0.455056	
yebY	b1839	3.529954		2.00959	
yecR	b1904	0.181591		0.234539	
yeeE	b2013	3.152955		1.38253	
yeeN	b1983	5.356315		2.833294	
yegE	b2067	0.210083		0.261364	
yegU	b2099	0.256246		0.519909	
yehK	b4541	0.30788		0.589756	
yejE	b2179	0.34779		0.467722	

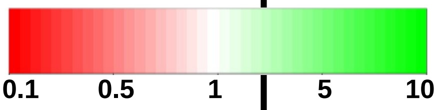

Table S8. (continued)

Gene	Accession ID	Mn F Change	Color Code	Mn+Fe F Change	Color Code
yfcE	b2300	7.004664		6.462052	
yfcG	b2302	0.272791		0.261568	
yfdL	b2355	0.34202		0.52773	
yfdM	b2356	0.351187		0.563945	
yfdY	b2377	0.103946		0.052087	
yfjM	b2629	0.306434		0.370818	
yfjY	b2644	0.126963		0.35996	
ygbA	b2732	3.976075		2.207343	
ygdB	b2824	0.259206		0.599721	
ygdD	b2807	0.245434		0.449451	
ygdH	b2795	0.350692		0.40257	
ygeF	b2850	0.166502		0.104716	
ygeV	b2869	0.179381		0.368801	
ygeW	b2870	0.240329		0.376407	
ygeY	b2872	0.304565		0.542631	
yggR	b2950	0.212623		0.518892	
yggU	b2953	3.105834		1.404168	
ygiQ	b4469	7.746334		2.968244	
ygjQ	b3086	0.320515		0.677252	
yhaL	b3107	5.069036		1.318979	
yhbE	b3184	3.829628		1.386921	
yhbV	b3159	0.228646		0.609453	
yhcB	b3233	4.830562		1.723446	
yhfS	b3376	0.178965		0.625086	
yhhY	b3441	0.266357		0.551936	
yhjK	b3529	4.473045		2.736628	
yiaN	b3578	0.341085		0.891264	
yibB	b3618	6.696972		1.876392	
yibN	b3611	3.237718		2.266261	
yicC	b3644	3.692614		1.983986	
yicO	b3664	0.185714		0.593164	
yicT	b4653	0.350974		0.913651	
yieP	b3755	0.332093		0.427163	
yihT	b3881	0.340448		0.938198	
yihV	b3883	0.320702		0.805546	
yjbJ	b4045	4.768225		8.109501	
yjcH	b4068	0.277908		0.796899	
yjcZ	b4110	0.096972		0.288767	
yjgL	b4253	0.115894		0.146858	
yjhE	b4282	0.305975		0.757814	
yjhX	b4566	0.278325		0.452805	
yjiK	b4333	0.247134		0.359646	
yjiL	b4334	0.181319		0.45498	
yjiM	b4335	0.208769		0.391232	
yjiS	b4341	0.262704		0.634836	
yjjI	b4380	0.262097		0.287439	
yjjJ	b4385	3.096381		1.912607	
ykfH	b4504	0.335842		0.619639	
ykfK	b4628	0.108623		0.33588	
ykfL	b4627	0.122963		0.319663	
ylbF	b0520	0.291672		0.54419	
ylcG	b4509	0.219739		0.431111	
ymfD	b1137	0.190511		0.143604	
ymfR	b1150	0.321977		0.646568	
ymgD	b1171	7.375248		1.553374	
ymgG	b1172	3.497752		1.559288	
ynfB	b1583	0.305754		0.278062	
ynfK	b1593	0.307573		0.31823	
yniA	b1725	0.299733		0.547587	
ynjA	b1753	0.192085		0.324613	
yohC	b2135	0.304339		0.497023	
yphB	b2544	0.159807		0.374958	
ypjJ	b4548	0.233404		0.501864	
ypjK	b2635	0.173224		0.435506	
yqeB	b2875	0.261615		0.470739	
yqeC	b2876	0.279733		0.639217	
yqgB	b2939	3.413245		1.583376	
yqgC	b2940	3.407138		1.661044	
yqiC	b3042	3.836802		4.522693	
ytfF	b4210	0.350513		0.779356	
ytfH	b4212	0.304594		0.611354	

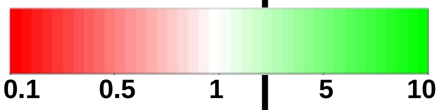

Supplement: Supplementary file 1 — Supplementary materials [file 41598_2017_12004_MOESM1_ESM.pdf]
